# Downregulated METTL14 accumulates BPTF that reinforces super-enhancers and distal lung metastasis via glycolytic reprogramming in renal cell carcinoma

**DOI:** 10.7150/thno.55424

**Published:** 2021-01-26

**Authors:** Chuanjie Zhang, Li Chen, Yihan Liu, Jingyi Huang, Ao Liu, Yang Xu, Yan Shen, Hongchao He, Danfeng Xu

**Affiliations:** 1 Department of Urology, Ruijin Hospital, Shanghai Jiao Tong University School of Medicine, Shanghai 200025, China.; 2 Department of Pharmacy, Shanghai Xuhui District Central Hospital, No. 966 Huaihai Middle Road, Xuhui District, Shanghai 200031, China.; 3 Department of Epidemiology and Biostatistics, School of Public Health, Nanjing Medical University, Nanjing 211166, China.; 4 Research Center for Experimental Medicine, Ruijin Hospital Affiliated to Shanghai Jiao Tong University School of Medicine, 197 Ruijin Road II, Shanghai, 200025, China.

**Keywords:** metastatic RCC, METTL14, BPTF, super-enhancers, glycolysis

## Abstract

**Background:** Methyltransferase-like 14 (METTL14) participates in tumorigenesis in several malignancies, but how METTL14 mediates the metastasis of renal cell carcinoma (RCC) has never been reported.

**Methods:** Western blotting, quantitative real-time PCR, and immunohistochemistry were used to determine the mRNA and protein levels of relevant genes. Methylated RNA immunoprecipitation sequencing and RNA sequencing were utilized to screen potential targets of METTL14. Chromatin immunoprecipitation sequencing and assay for transposase-accessible chromatin sequencing were performed to investigate epigenetic alterations. The biological roles and mechanisms of METTL14/BPTF in promoting lung metastasis were confirmed *in vitro* and *in vivo* using cell lines, patient samples, xenograft models, and organoids.

**Results:** Utilizing the TCGA-KIRC and Ruijin-RCC datasets, we found low expression of METTL14 in mRCC samples, which predicted poor prognosis. METTL14 deficiency promoted RCC metastasis *in vitro* and *in vivo*. Mechanistically, METTL14-mediated m^6^A modification negatively regulated the mRNA stability of bromodomain PHD finger transcription factor (BPTF) and depended on BPTF to drive lung metastasis. Accumulated BPTF in METTL14-deficient cells remodeled the enhancer landscape to reinforce several oncogenic crosstalk. Particularly, BPTF constituted super-enhancers that activate downstream targets like enolase 2 and SRC proto-oncogene nonreceptor tyrosine kinase, leading to glycolytic reprogramming of METTL14^-/-^ cells. Finally, we determined the efficacy of the BPTF inhibitor AU1 in suppressing mRCC of patient-derived cells, mRCC-derived organoids (MDOs), and orthotopic xenograft models.

**Conclusions:** Our study is the first to investigate the essential role of m^6^A modification and the METTL14/BPTF axis in the epigenetic and metabolic remodeling of mRCC, highlighting AU1 as a vital therapeutic candidate.

## Introduction

Kidney cancer is one of the most common malignancies in the urology system and accounts for ~3% of all adult malignancies worldwide 1. Currently, the main treatment for managing renal cell carcinoma (RCC) is laparoscopic surgical resection followed by immunotherapy or targeted therapy, such as sunitinib 2-4. However, many RCC patients are first diagnosed at advanced stages, and overall prognosis is poor 5. In more than 30% of RCC patients, the disease inevitably progresses to a metastasized terminal state that tends to be resistant to multiple drugs 6. As the most hazardous event, tumor metastasis determines the treatment strategy and overall prognosis for RCC patients. Therefore, identification of novel biomarkers and mechanisms underlying the metastatic and progressive features of RCC is urgently needed.

Recently, *N*^6^-methyladenosine (m^6^A) methylation has been found to play important roles in diverse biological processes, including immunity, metabolism, stemness maintenance, and tumor differentiation 7-9. The level of m^6^A modification is dynamically regulated by methyltransferase complexes (writers) and m^6^A demethylases (erasers). In addition to mRNA, some noncoding RNAs (tRNA, rRNA, lncRNA, and snRNA) were found to be regulated during the m^6^A modification process, indicating the vital function of this modification in transcription-level control 9-11. The common members of the methyltransferase complex include METTL3, METTL14, and WTAP, whereas FTO and ALKBH5 possess m^6^A demethylase activity. Other m^6^A readers, including YTHDF1, YTHDF2, YTHDF3, and YTHDC1, also determine RNA stability 12, 13. Intriguingly, we observed a distinct oncogenic influence of several m^6^A RNA enzymes in different tumors 14. In particular, METTL14, targeting MYB and MYC, was proven to be required for the growth of acute myeloid leukemia cells, driving the self-renewal process of leukemia stem/initiation cells 15. Nevertheless, METTL14 may significantly suppress the development and metastasis of colorectal cancer by targeting the oncogenic long noncoding RNA XIST 16. Furthermore, we observed that METTL14 expression levels were remarkably downregulated in RCC, especially in samples at advanced metastatic stages. To date, little has been reported about the specific biological role of METTL14 in RCC, and we intend to discover the possible associations between aberrant METTL14 levels and metastatic RCC.

Dysregulated epigenetic machinery, consisting of mutated or overexpressed chromatin remodelers and modifiers, has been recognized as a determinant for lineage plasticity, tumorigenesis, and the malignant progression of multiple tumor cells 17-19. The nucleosome remodeling factor (NURF) complex, which is common to eukaryotic species, facilitates the dynamic transcription process mediated by chromatin in an ATP-dependent manner 20, 21. The bromodomain PHD finger transcription factor (BPTF), constituting the largest subunit of the NURF complex, was reported to participate in tumor progression mainly by regulating DNA accessibility and gene expression 22. BPTF was reported to maintain stemness traits and regulate the growth of adult and pediatric high-grade glioma by targeting the MYC pathway 23. In particular, high-throughput sequencing data indicated that BPFT expression levels correlated significantly with epithelial-mesenchymal transition (EMT) markers in colorectal carcinoma and promoted cancer cell migration in triple-negative breast cancer 24-26. Despite the significant role of BPTF in mediating the progression and metastasis of tumors, few studies have addressed the transcriptional or posttranscriptional regulation of BPTF. In addition, the specific oncogenic role of BPTF in RCC evolution, especially during metastatic events, remains unclear. Whether m^6^A modification could influence BPTF stability to alter epigenetic remodeling or contribute to the malignant features of RCC is unclear and worthy of investigation.

In the current study, we observed that m^6^A modification was less extensive and METTL14 was significantly downregulated in metastatic RCC samples. We demonstrated that low METTL14 enhanced BPTF stability, which drove RCC metastasis *in vitro* and *in vivo*. Accumulated BPTF in METTL14^-/- or low^ RCC cells reinforced super-enhancers to mainly trigger the aerobic glycolysis pathway. Considering these findings, we have provided novel insights into METTL14-mediated m^6^A modification during chromatin remodeling and promising targets to efficiently suppress RCC metastasis.

## Materials and Methods

### Specimens and cell culture

This research was approved by the Ethics Committee of Ruijin Hospital, School of Medicine, Shanghai Jiao Tong University. RCC specimens (N = 280) were histopathologically confirmed by three independent pathologists and derived with informed consent. The RCC samples were made into a tissue microarray with matched normal sections. We also obtained RCC lung metastases via computed tomography (CT)-guided biopsy. Patient biopsies and resections were placed in a sterile conical tube containing ACL-4 medium (Invitrogen) with 10% fetal bovine serum (FBS) and 1% antibiotic-antimycotic (Fisher Scientific) and kept on wet ice during transport from the operating room to the research laboratory. The patient biopsies were enzymatically digested with 25 mg/mL liberase in 5 mL ACL-4 medium for 1h in a 37°C MultiTherm shaker (Benchmark Scientific) set at 1000 rpm. The resections were digested using a Tumor Dissociation Kit (Miltenyi Biotec) and gentleMACS Dissociators with heating. Dissociation was stopped by adding 0.5 mL FBS. After digestion or lysis, cells were separated into a number of plating conditions dependent on tissue pellet size. These conditions included collagen-coated dishes with RPMI and 10% FBS (R10), DMEM and 10% FBS (D10), or ACL-4 and FBS (A<5 and AR5) media.

Renca cells, a murine renal carcinoma cell line, and human ccRCC cell lines (786-O, ACHN, Caki-1) were purchased from ATCC or Cell Bank, Shanghai Institute of Biochemistry and Cell Biology, Chinese Academy of Sciences. The cells were cultured at 37 °C in an atmosphere of 5% CO_2_ and respectively maintained in RPMI 1640 medium (Gibco-BRL) supplemented with 10-15% FBS (Invitrogen) or keratinocyte‐SFM (Invitrogen) with 100 U/mL penicillin and 100 μg/mL streptomycin.

### Stable gene knockout cells generation by CRISPR/Cas9

The plasmid pX459 was utilized to clone guide oligos targeting METTL14 or BPTF. The designed gene-specific sgRNAs are listed in Table S1. 786-O cells were plated and transfected with pX459 constructs overnight, then screened with 1 μg/mL puromycin for three days. Monoclonal cell lines were isolated by seeding the remaining cells in 96-well plates. The knockout efficiency of the cell clones was determined by western blot or Sanger sequencing.

### Quantitative real-time reverse transcription polymerase chain reaction (qRT-PCR)

Total RNA was extracted from cells using TRIzol reagent (Tiangen), and cDNA was reverse-transcribed using the Superscript RT kit (TOYOBO) following the manufacturer's instructions. The SYBR Green PCR Master Mix kit (TOYOBO) was used to perform PCR amplification. All quantitations were normalized to endogenous GAPDH expression levels. The sequences of the primers used for qRT-PCR are listed in Table S1.

### Methylated RNA immunoprecipitation sequencing (MeRIP-seq)

Total RNA was extracted from transfected 786-O cells, then mRNA sequencing and m^6^A sequencing were performed simultaneously (Jiayin Biomedical Technology). For mRNA sequencing, mRNAs were single-end sequenced with Illumina HiSeq 2000. Transcript assembly and differential expression was evaluated by Cufflinks with Refseq mRNAs to guide assembly. For m^6^A sequencing, mRNA was fragmented and then incubated with m^6^A antibody (New England BioLabs) for immunoprecipitation. Immunoprecipitated RNA was analyzed by qRT-PCR or high-throughput sequencing.

### Epigenetic analyses

Chromatin immunoprecipitation sequencing (ChIP-seq) was performed using an Assay Kit (Millipore) according to the manufacturer's instructions. Cross-linked chromatin was sonicated into fragments, then the fragments were immunoprecipitated with indicated antibodies.

Assay for transposase-accessible chromatin sequencing (ATAC-seq) was performed by precipitating 5 × 10^5^ METTL14^-/-^ cells with or without BPTF ablation. The precipitated cells were kept on ice and subsequently resuspended in 25 mL 2× TD Buffer (Nextera kit, Illumina), 2.5 mL transposase enzyme (Nextera kit, Illumina) and 22.5 mL nuclease-free water in a total reaction volume of 50 mL for 1 h at 37 °C. DNA was then purified using MinElute PCR Purification Kit (Qiagen) in a final volume of 10 mL. ATAC-seq libraries were sequenced as 50 base paired-end reads on a HiSeq 4000 (Illumina) at Jiayin Biomedical Technology, Shanghai, China.

### RNA stability assays

RCC cells were seeded in 12-well plates overnight, and then treated with 5 μg/mL actinomycin D (MedChemExpress) at 0, 2, 4, and 6 h. TRIzol (Invitrogen) was utilized to isolate the total RNA and the results were analyzed by qPCR. The mRNA expression for each group at each time point was calculated and normalized to β-actin.

### Western blot analysis and RNA immunoprecipitation

Transfected cells were incubated in lysis buffer (50 mM Tris-HCl, pH 7.5, 150 mM NaCl, 15 mM MgCl_2_, 5 mM EDTA, 0.1% NP-40) containing a protease inhibitor cocktail (Roche) for subsequent co-immunoprecipitation. The beads were separated and washed with cold phosphate-buffered saline (PBS), and then subjected to western blot analysis. For the western blotting, equivalent amounts of protein were separated by SDS-PAGE at 80 V for 2.5 h and transfected to PVDF membranes for 2 h. The membranes were incubated with specific antibodies for METTL14 (Cell Signaling Technology), BPTF (Abcam), E-cadherin (Cell Signaling Technology), N-cadherin (Cell Signaling Technology), vimentin (Cell Signaling Technology), enolase 2 (ENO2; Cell Signaling Technology), SRC proto-oncogene nonreceptor tyrosine kinase (SRC; Cell Signaling Technology), and β-actin (Abcam) at 4 °C overnight, washed with 1% TBST for 30 min, then incubated with secondary antibodies (1:5000, BioTNT) for 2 h. Finally, they were washed with 1% TBST and detected by a chemiluminescence system (Amersham Biosciences).

### Immunofluorescence and confocal microscopy

Cells were plated on chamber slides then fixed with 4% paraformaldehyde at room temperature for 30 min. After washing with PBS, the cells were permeabilized with 0.1% Triton X-100 in PBS for 15 min at room temperature. The cells were then washed with PBS with Tween 20 (PBST), blocked with 5% donkey serum in PBS for 1 h, and incubated with primary antibodies in PBS overnight at 4 °C in the dark. After washing with PBST, fluorophore-labelled secondary antibodies were applied and the cells were counterstained with DAPI for 1 h at room temperature in the dark. The slides were mounted in ProlongGold (Invitrogen). The cells were visualized and imaged using an LSM710 confocal microscope (Zeiss) with a 63× 1.4 NA oil PSF objective. The analysis results were collected in triplicate from three different fields of view.

### Migration and invasion assays

Cell migration ability was assessed by a wound-healing assay. The details are provided in Supplementary Information. Briefly, an artificial wound was created in 786-O and ACHN cells 24 h after transfection using a 200 μL pipette tip. The cells were then cultured with 2% FBS for 20 h. To visualize the migrated cells and wound healing, images were taken at 0 and 20 h after the wound was created. To analyze the invasive ability of the cells, 24-well Transwell plates with 8 µm pore polycarbonate membrane inserts (Corning) were used according to the manufacturer's protocol. The membrane was coated with 200 ng/mL Matrigel (BD Biosciences). After 20 h of incubation, cells invading into the lower surface of the membrane insert were fixed in 4% paraformaldehyde, stained with 0.5% crystal violet, and quantified by counting 10 random fields of view.

### Establishment and culture of mRCC-derived organoids (MDOs)

CT-guided biopsies were conducted to obtain mRCC lung metastases. The biopsies were conditioned in 5 mL PBS with 5 mM EDTA for 15 min at room temperature, then digested in 5 mL PBS with 1 mM EDTA and 2× TrypLe (Thermo Fisher Scientific) for 1 h at 37 °C. Following digestion, mechanical force (pipetting) was applied to facilitate cell release in solution. Dissociated cells were collected in Advanced DMEM/F-12 (Thermo Fisher Scientific), pelleted, resuspended in 120 µL growth factor reduced (GFR) Matrigel (Corning), and seeded in 24- or 48-well flat bottom cell culture plates (Corning). The Matrigel was then solidified for 20 min in a 37° C and 5% CO_2_ cell culture incubator, and overlaid with 500 µL complete human organoid media. The complete media was refreshed every two days. Passaging of MDOs was performed using TrypLe. Briefly, MDOs were mechanically harvested (by pipetting) out of Matrigel using PBS with 1 mM EDTA and 1× TrypLe, then incubated for 20 min at 37 °C. MDOs were then dissociated to single cells by applying mechanical force (pipetting), washed with HBSS (Thermo Fisher Scientific), pelleted, resuspended in GFR Matrigel, and re-seeded at an appropriate ratio. Additionally, specific human MDO culture media were collected, which were made up of the specific constituents shown in Table S1.

### Animal studies

Male athymic BALB/c nude mice aged 4 weeks were used to generate an *in vivo* lung metastasis model. ACHN, 786-O, or MDO cells stably expressing luciferase were injected into the flanks and tail vein. Lung metastatic progression was monitored and imaged using an IVIS-100 system (Caliper Life Sciences) 10 min after intraperitoneal injection of 300 mg/kg VivoGlo™ Luciferin (Promega) in 80 μL of saline. H&E staining and immunohistochemical analysis were performed to assess the sizes of the metastases. To generate a highly metastatic orthotopic xenograft model, 1 × 10^5^ luciferase-expressing Renca cells (Luc-Renca) in 25 µL of 2:1 (v/v) PBS:Matrigel were injected into the right sub-renal capsule of the kidney of BALB/c mice (4 mice/group). *In vivo* bioluminescence imaging (BLI) was done every 7 days. Living Image® software was used to analyze the data. Mice were euthanized 28 days after implantation, and lung metastatic lesions were analyzed using the BLI system.

### Statistical analysis

All data and error bars are mean ± standard deviation from at least three independent experiments. Student's *t*-test and one-way ANOVA were used to compare differences with continuous variables. Fisher's exact test and chi-squared test were used to deal with categorical variables. Kaplan-Meier method was used to draw the survival plots based on the log-rank test. Univariate and multivariate Cox regression analyses were performed to assess independent factors. Pearson's test was used to determine correlations between different groups. All statistical analyses were conducted in R studio (version 3.5.3). The indicated *P* values (*P < 0.05, **P < 0.01, ***P < 0.001) were regarded to be statistically significant.

## Results

### METTL14 expression is low in RCC and correlated with clinical metastasis and prognosis

**Figure 1:**
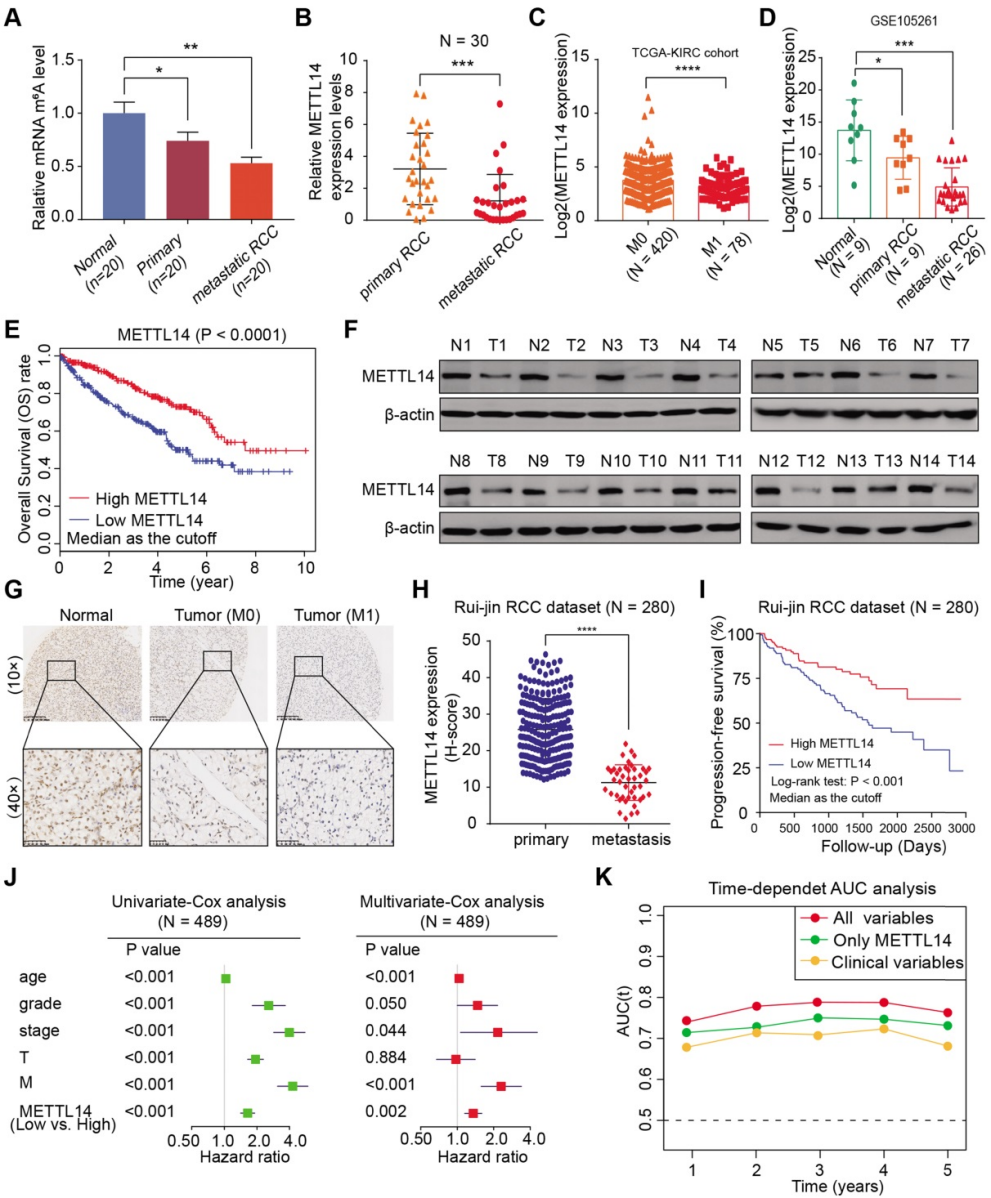
** METTL14 is downregulated and associated with tumor metastasis and prognosis in RCC patients. (A)** Global m^6^A levels across normal, primary RCC, and mRCC cells. **(B)** mRNA levels of METTL14 in 30 paired RCC tissues from Ruijin hospital. **(C)** METTL14 expression in primary RCC and mRCC samples in the TCGA-KIRC dataset. **(D)** METTL14 expression in normal, primary RCC, and mRCC samples in the GSE105261 dataset. **(E)** Kaplan-Meier analysis of OS stratified by METTL14 expression in the TCGA-KIRC dataset. **(F)** Western blots of METTL14 protein expression in paired normal (N) and tumor (T) samples from the Ruijin-RCC dataset. **(G)** Representative images of IHC staining for METTL14 protein in the Ruijin-RCC tissue array. **(H)** METTL14 protein expression in primary RCC and mRCC in the Ruijin-RCC dataset. **(I)** Kaplan-Meier analysis of PFS stratified by METTL14 protein expression in the Ruijin-RCC dataset. **(J)** Uni- and Multi-variate analyses of the TCGA-KIRC cohort. **(K)** Time-dependent receiver operating characteristic analysis comparing the predictive efficiency of METTL14 level alone and in combination with other clinical variables.

Although emerging studies have proposed that m^6^A plays essential roles in many biological processes, including cancer progression, related investigations into kidney cancer have been scarce. To systematically investigate the underlying roles of m^6^A modifications in RCC, we first measured the m^6^A modification levels in normal kidney cells, primary tumor cells, and lung metastatic cells from biopsy samples. We utilized an m^6^A RNA methylation quantification kit to detect global m^6^A levels among cultured cells from tissues and observed remarkably decreased global m^6^A levels in metastatic RCC cells, differing from colon cancer, melanoma, and leukemia (Figure 1A). Given that the m^6^A modification is mostly regulated by common m^6^A writers (METTL3/14, WTAP) and erasers (ALKBH5, FTO), we conducted a comprehensive bioinformatics analysis and found that METTL14 was expressed at significantly lower levels in The Cancer Genome Atlas Kidney Renal Clear Cell Carcinoma (TCGA-KIRC) and International Cancer Genome Consortium (ICGC)-RCC cohorts, compared with other enzymes (Figure S1A). We further detected the mRNA levels of METTL14 in 30 paired RCC primary and metastatic samples and observed that METTL14 was remarkably decreased in the metastatic samples, while the expressions of other enzymes were not significantly different (Figure 1B, Figure S1B). Additionally, compared with control cells, METTL14 deficiency resulted in remarkably decreased m^6^A modification levels, suggesting the critical role of METTL14 in m^6^A modification of RCC (Figure S1C). In addition, METTL14 expression levels were found to be negatively correlated with metastasis in TCGA-KIRC and GSE105261 dataset cohorts 27 (Figure 1C-D). Kaplan-Meier analysis also verified that patients with lower METTL14 levels showed a worse overall survival (OS) prognosis (log-rank test, *P* < 0.0001, Figure 1E). Consistent with these results, we also detected notably lower METTL14 protein levels in fresh tumor samples than in paired normal tissues, and this low expression level was found in 78.6% (11/14) of the samples, as determined by western blot analysis (Figure 1F).

We confirmed these findings in an independent dataset, Ruijin-RCC (N = 280), using IHC of a tissue microarray. We first observed significantly lower protein levels of METTL14 in the mRCC samples than in the primary RCC or normal tissues (Figure 1G). A correlation analysis further corroborated that METTL14 levels were negatively associated with metastatic stage (*P* < 0.001, Figure 1H). Moreover, Kaplan-Meier analysis of the Ruijin-RCC dataset suggested that patients with lower METTL14 levels had worse 3-year progression-free survival (PFS) outcomes (log-rank test, *P* < 0.0001, Figure 1I). In addition, we conducted a univariate Cox analysis of the TCGA-KIRC cohort and found that age, tumor grade, pathological stage, T stage, metastatic status, and METTL14 level correlated significantly with 5-year OS (Figure 1J). Multivariate Cox analysis also revealed that low METTL14 level was an independent risk factor, in contrast to other clinical hazard factors (HR = 1.541, *P* = 0.002, Figure 1J). Finally, we performed a time-dependent receiver operating characteristic curve analysis and found that its predictive efficiency was superior when METTL14 levels were combined with other clinical variables than when it was based on either METTL14 levels or clinical variables separately (Figure 1K). The clinical baseline characteristics of RCC patients in this study are summarized in Table S2. Taken together, these results indicate that m^6^A modification and METTL14 expression are significantly lower in metastatic RCC tumors than in primary tissues, and METTL14 possesses the potential to be an independent biomarker for predicting prognosis and metastasis in RCC.

### METTL14 correlates negatively with RCC metastasis and EMT processes *in vitro* and *in vivo*

**Figure 2:**
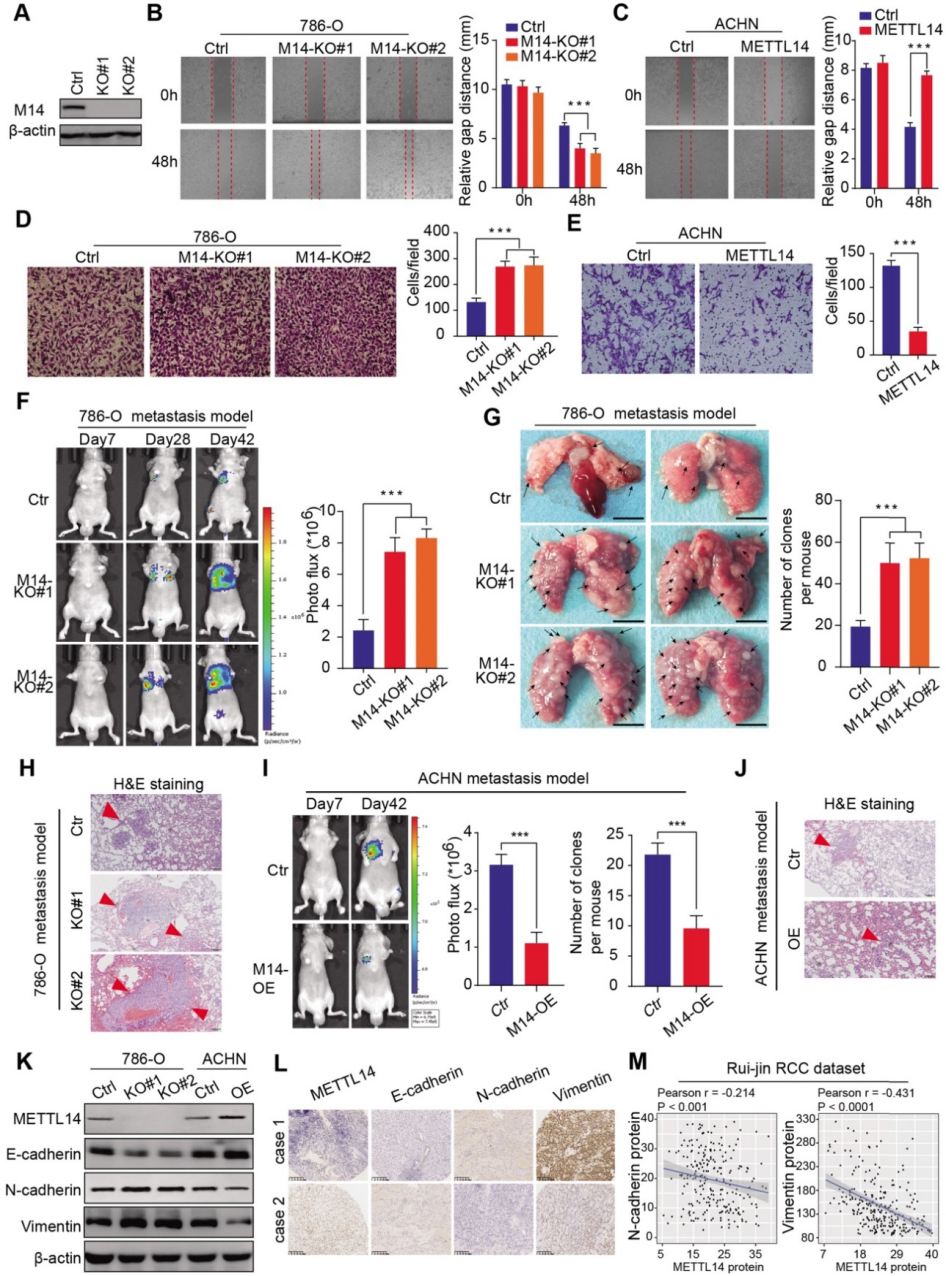
** METTL14 deficiency promoted RCC metastasis *in vitro* and *in vivo* and correlated with EMT processes. (A)** Western blot of METTL14 knockout efficiency in 786-O cells. **(B-E)** Wound healing, migration, and invasion assays of WT and METTL14^-/-^ (M14-KO#1 and M14-KO#2) 786-O cells and WT and METTL14-overexpressing (M14-OE) ACHN cells. **(F-H)** *In vivo* BLI, white-light images of excised lungs, and H&E staining of a metastasis model generated with WT and METTL14^-/-^ Luc-786-O cells. **(I-J)** *In vivo* BLI and H&E staining of a metastasis model generated with WT and M14-OE Luc-ACHN cells. **(K)** Western blots of the protein expressions of METTL14 and EMT markers in the RCC cell lines. **(L-M)** Representative IHC staining and correlation analyses of METTL14 and EMT markers in RCC samples.

Given that METTL14 levels correlated significantly with clinical NM stages in the RCC samples, we conducted experimental assays to determine the role of METTL14 in metastatic RCC. METTL14 knockout significantly promoted the migration speed of RCC cells, while METTL14 overexpression remarkably suppressed their migratory ability (Figure 2A-C, Figure S2A). In addition, a Transwell Matrigel invasion assay confirmed that the invasive ability of RCC cells was markedly increased in response to METTL14 ablation but was suppressed upon forced expression of METTL14 in two cell lines (Figure 2D-E, Figure S2B-C). Moreover, ectopic expression of METTL14 completely restrained the enhanced migratory and invasive ability of RCC cells (786-O and ACHN) induced by METTL14 knockdown (Figure S2D). In addition, *in vivo* assays were performed to further evaluate the effects of METTL14 on RCC metastasis. We generated stable luciferase-labeled cells with modified METTL14 that were then injected into the tail vein of BALB/c nude mice. We measured the luciferase signals at different time points to monitor the tumor location and metastasis into the lung. After 56 days, we observed that METTL14 knockout remarkably increased the metastatic ability of RCC cells into the lung compared to that of the control group, as quantified by BLI and the number and size of lung metastases (Figure 2F-H). In contrast, METTL14 overexpression significantly impeded metastatic processes compared with the control group in an ACHN metastasis model, as indicated by the number and size of lung metastatic lesions (Figure 2I-J, Figure S2E). Finally, we further explored the underlying relationships between METTL14 levels and EMT markers. As shown in Figure 2K, METTL14 deficiency in RCC cell lines remarkably promoted the EMT process with upregulated N-cadherin and vimentin and downregulated E-cadherin. However, METTL14 overexpression resulted in a significant decrease in N-cadherin and vimentin along with an increase in E-cadherin in ACHN cells (Figure 2K). Accordingly, we used two representative RCC specimens with relatively high and low METTL14 levels to confirm these results. IHC data indicated that METTL14 proteins were negatively correlated with the levels of EMT markers (Figure 2L-M). Collectively, these findings suggest that METTL14 deficiency promotes RCC metastasis *in vitro* and *in vivo* and additionally correlates with EMT processes.

### BPTF mRNA stability is negatively regulated by METTL14-mediated m^6^A modification

**Figure 3:**
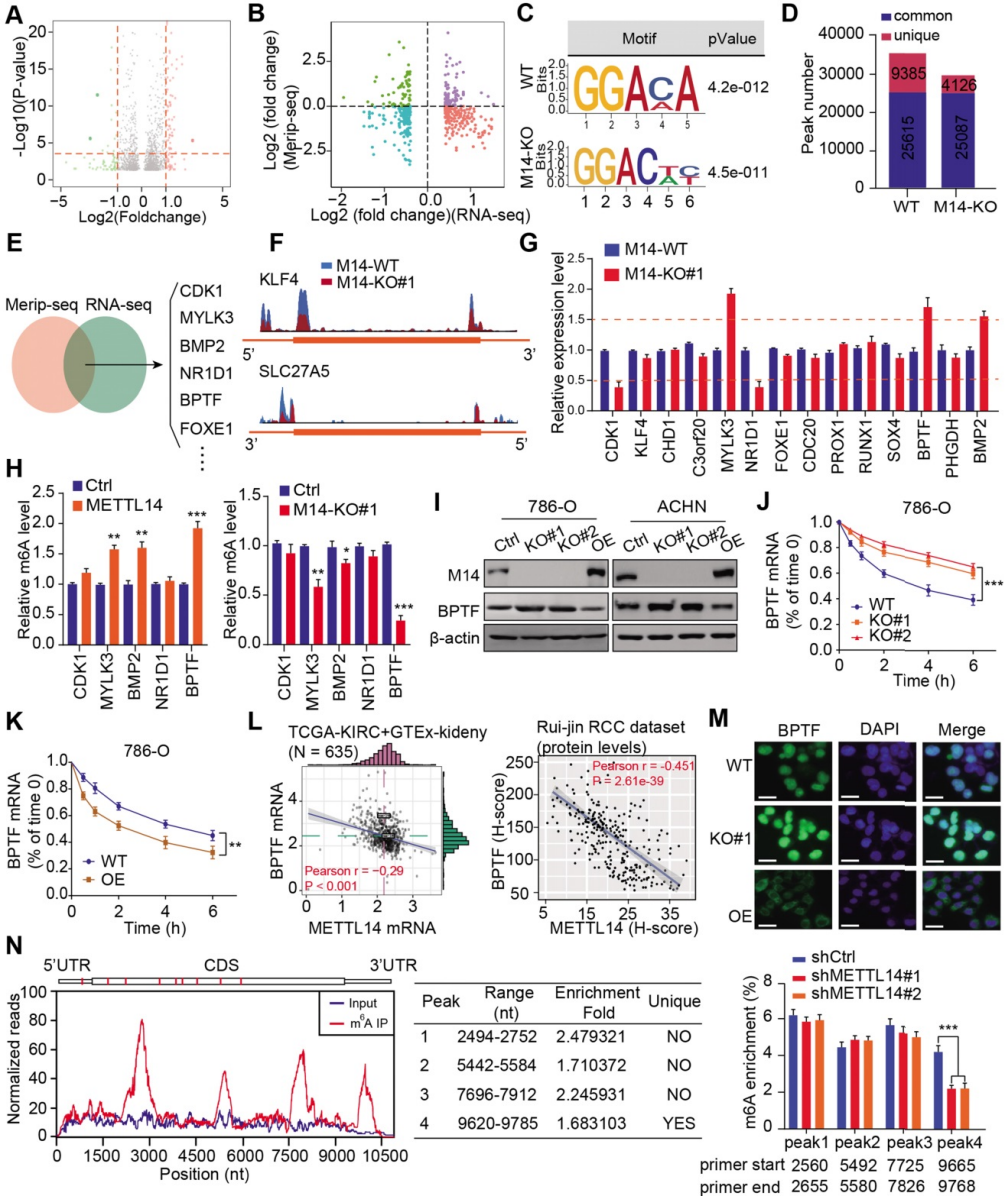
** Screening and validation of BPTF as the METTL14 downstream target by MeRIP-seq and RNA-seq. (A)** Volcano plot illustrating the distributions of DEGs in WT and METTL14^-/-^ (M14-KO#1) cells. **(B)** Quadrant chart of the distribution of peaks with a notable change in both m^6^A level and RNA expression. **(C)** Top consensus motif recognized by HOMER with m^6^A-seq peaks in 786-O cells with or without METTL14 knockout. **(D)** m^6^A-seq peak counts in WT and METTL14^-/-^ cells. **(E-F)** Combination of MeRIP-seq and RNA-seq data identifying potential candidates and m^6^A tracks in representative targets.** (G)** mRNA alterations upon METTL14 deficiency in 14 candidates determined by qPCR. **(H)** METTL14-dependent alterations in the m^6^A levels of representative genes based on gene-specific m^6^A qPCR analysis. **(I)** Western blot assay of METTL14 and BPTF protein levels in the RCC cell lines.** (J-K)** METTL14-dependent BPTF stability in 786-O cells treated with actinomycin D. **(L)** Relationships between METTL14 and BPTF mRNA and protein levels in the TCGA-KIRC and GTEx-kidney cohorts and the Ruijin-RCC dataset.** (M)** Confocal microscopy images of METTL14-dependent BPTF localization and expression. Scale bar = 20 μm. **(N)** Left: relative abundance of m^6^A sites along BPTF mRNA in 786-O cells. The blue and red reads are from the non-IP control input and MeRIP of 786-O cells, respectively. Middle: identification of the unique METTL14-mediated peak on BPTF-3'UTR. Right: MeRIP-qPCR analysis of peak region enrichment in fragmented BPTF RNA.

To investigate the role of METTL14 in RCC, we performed RNA-seq to compare the differential expression profiles of wildtype (WT) and METTL14^-/-^ 786-O cells. A total of 742 differentially expressed genes (DEGs) were identified based on a cutoff of |fold change| > 1 and *P* < 0.01 (Figure 3A, Table S3). Subsequent Gene Ontology (GO) analysis indicated that the DEGs were mainly enriched in the cell cycle, cellular assembly and organization, small molecule catabolic, and DNA replication and recombination processes (Figure S3A), implicating an oncogenic role for METTL14 in RCC. To screen the putative targets that were altered by METTL14-mediated m^6^A modification, we combined MeRIP-seq data with RNA-seq data to illustrate the distribution of peaks with notable changes at both the RNA and m^6^A levels. Specifically, we found 362 hypomethylated m^6^A peaks with higher mRNA expression in METTL14-knockout cells than in control cells, and these peaks were identified as METTL14-related hypo-upregulation peaks (Figure 3B). The RRACH motif (R = G or A and H = A, C, or U) has been associated with m6A recognition, and in our study, the consensus GGAC motif was found to be enriched in immunopurified RNA in the control 786-O cells (Figure 3C). In addition, the m^6^A-seq data was screened, and the results showed 35,000 and 29,213 peaks from 14,718 and 15,292 m^6^A-modified transcripts in the control and METTL14-deficient cells, respectively (Figure 3D). We thus observed 4126 new peaks that emerged with the elimination of 9385 peaks. In addition, we further overlapped the MeRIP-seq and RNA-seq data to identify potential candidates and illustrated the m^6^A tracks to representative targets (Figure 3E-F). After filtering the tumor-unrelated genes and the ones with rarely reported associations with tumors, we finally obtained 14 relevant genes for further validation. MeRIP-qPCR revealed that 5 candidates, CDK1, MYLK3, NR1D1, BPTF, and BMP2, exhibited greater alteration upon METTL14 ablation compared with the other candidates (Figure 3G). We also conducted a gene-specific m^6^A pull-down assay to determine the m^6^A levels of the 5 genes and found that BPTF showed the most consistent alterations in m^6^A levels upon METTL14 overexpression or deficiency (Figure 3H). Recently, extensive evidences have suggested that aberrant BPTF may contribute to tumorigenesis in several malignancies. Collectively, given that little was known about the regulatory functions of BPTF in RCC, we selected BPTF as an essential target of METTL14-mediated m^6^A modification for subsequent investigation. Next, BPTF protein levels were verified to be negatively associated with METTL14 in RCC cell lines via western blot (Figure 3I). The RCC cells were accordingly treated with actinomycin D, an inhibitor of transcription, to determine whether m^6^A modification could influence BPTF mRNA stability. The BPTF mRNA levels were more stable in METTL14-knockout cells than in control cells, whereas METTL14 overexpression resulted in the opposite results in RCC cells (Figure 3J-K, Figure S3B). Additionally, we obtained the RNA-seq data of 635 samples (normal: 100, tumor: 535) from the TCGA-KIRC cohort and the Genotype-Tissue Expression (GTEx)-kidney dataset and 280 microarray samples from the Ruijin-RCC dataset. Correlation analysis confirmed the significantly negative relationships between BPTF and METTL14 levels at both the mRNA and protein levels (*P* < 0.0001) (Figure 3L). Based on the robust regulation between BPTF and METTL14, a confocal assay further directly revealed that BPTF was exclusively localized in the nucleus and enhanced upon METTL14 deficiency (Figure 3M). The corresponding western blot assay further confirmed these results (Figure S3C). Moreover, we also recognized four statistically significant m^6^A peaks in BPTF mRNA, especially one METTL14-mediated unique peak in the 3' untranslated region (UTR) (Figure 3N). To deeply investigate the significance of the BPTF-3'UTR, we constructed the whole BPTF transcript containing the coding sequence (CDS) with the 3'UTR (CDS-3'UTR) or the 3'UTR alone. After knocking down METTL14 with small interfering RNAs (siRNAs), we observed that FLAG-BPTF expression was notably increased in the 786-O cells transfected with the CDS-3'UTR construct but not CDS alone, suggesting that the 3'UTR played a vital role in METTL14-BPTF regulation (Figure S3D-E). Finally, we also performed a BPTF-3'UTR reporter luciferase assay and found that targeting METTL14 via specific siRNAs significantly enhanced the luciferase activity of the BPTF-WT-3'UTR construct, whereas it had no effect on the MUT-3'UTR construct with mutations at the unique peak sites (Figure S3F). Taken together, these data suggest that BPTF mRNA stability is negatively regulated by METTL14-mediated m^6^A modification at the 3'UTR region.

### METTL14 deficiency accelerates RCC metastasis by upregulating BPTF expression

**Figure 4:**
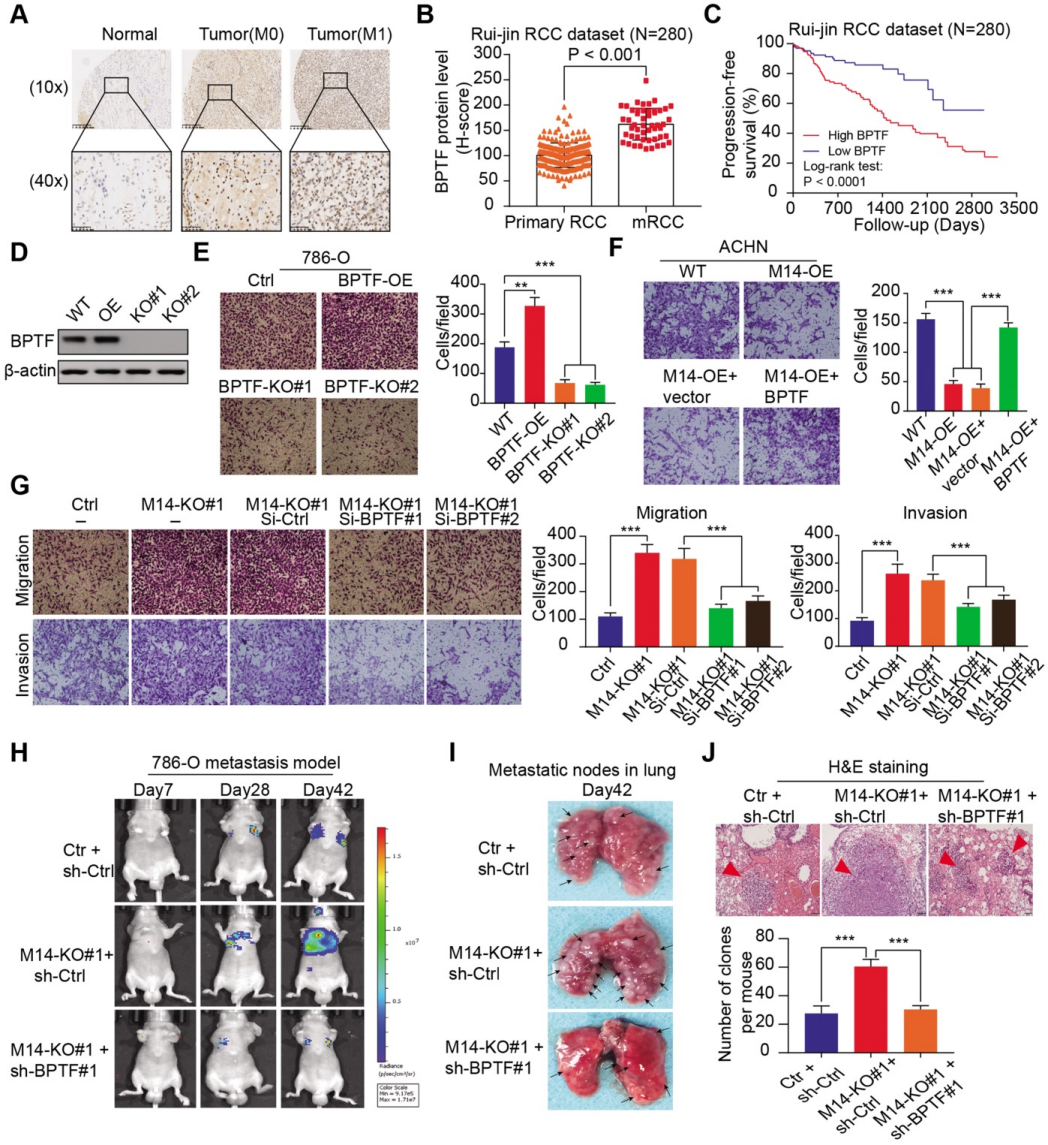
** METTL14 deficiency accelerates RCC metastatic phenotype by upregulating BPTF. (A-B)** Representative IHC images and quantitation of BPTF protein expression in normal, primary RCC, and mRCC tissues in the Ruijin-RCC dataset. **(C)** Kaplan-Meier analysis of PFS stratified by BPTF protein expression in the Ruijin-RCC dataset. **(D)** Western blot of BPTF knockout efficiency via sgRNAs in 786-O cells.** (E)** Migration assay of WT, BPTF^-/-^ (BPTF-KO#1 and BPTF-KO#2), and BPTF-overexpressing (BPTF-OE) 786-O cells. **(F)** Invasion assay of WT and METTL14-overexpressing (M14-OE) ACHN cells with and without transfection of BPTF. **(G)** Migration and invasion assays of METTL14^-/-^ 786-O cells transfected with BPTF siRNAs and related controls. **(H-J)** *In vivo* BLI, white-light images of excised lungs, and H&E staining of a metastasis model (N = 6) generated with WT and METTL14^-/-^ Luc-786-O cells with and without BPTF knockdown by shRNA.

Given that BPTF was identified as the *bona fide* target of METTL14, we sought to further characterize the oncogenic role of BPTF in RCC metastasis. In accordance with our speculations, BPTF was highly expressed in tumor samples, especially in metastatic cases, with *P* < 0.001 (Figure 4A-B). The Kaplan-Meier analysis of the Ruijin-RCC dataset indicated that patients with high BPTF expression showed worse PFS outcomes than those with low BPTF expression (log-rank test, *P* < 0.0001, Figure 4C). CRISPR/Cas9-mediated knockout of BPTF via sgRNA constructs or BPTF overexpression was verified by western blot analysis (Figure 4D). The results showed that BPTF deficiency remarkably impaired RCC migration (Figure 4E). In contrast, BPTF overexpression notably promoted RCC metastasis *in vitro* and *in vivo* compared with control cells (Figure 4F, Figure S3H). In addition, transient transfection of BPTF further rescued the inhibitory effect of METTL14 overexpression on RCC migration (Figure 4F). As expected, BPTF expression was efficiently knocked down using siRNAs, which significantly suppressed the migration and invasion induced by METTL14 deficiency in 786-O cells (Figure 4G). Furthermore, we constructed an *in vivo* metastatic model and observed that BPTF knockdown significantly suppressed the tumor metastatic ability of METTL14^-/-^ cells, which were quantified and compared by the number or size of lung metastatic lesions (Figure 4H-J, Figure S3G). Taken together, these findings suggest that low METTL14 may promote RCC metastasis through the upregulation of BPTF.

### Accumulated BPTF alters the oncogenic transcriptome and remodels the enhancer landscape in METTL14^-/-or low^ cells

**Figure 5:**
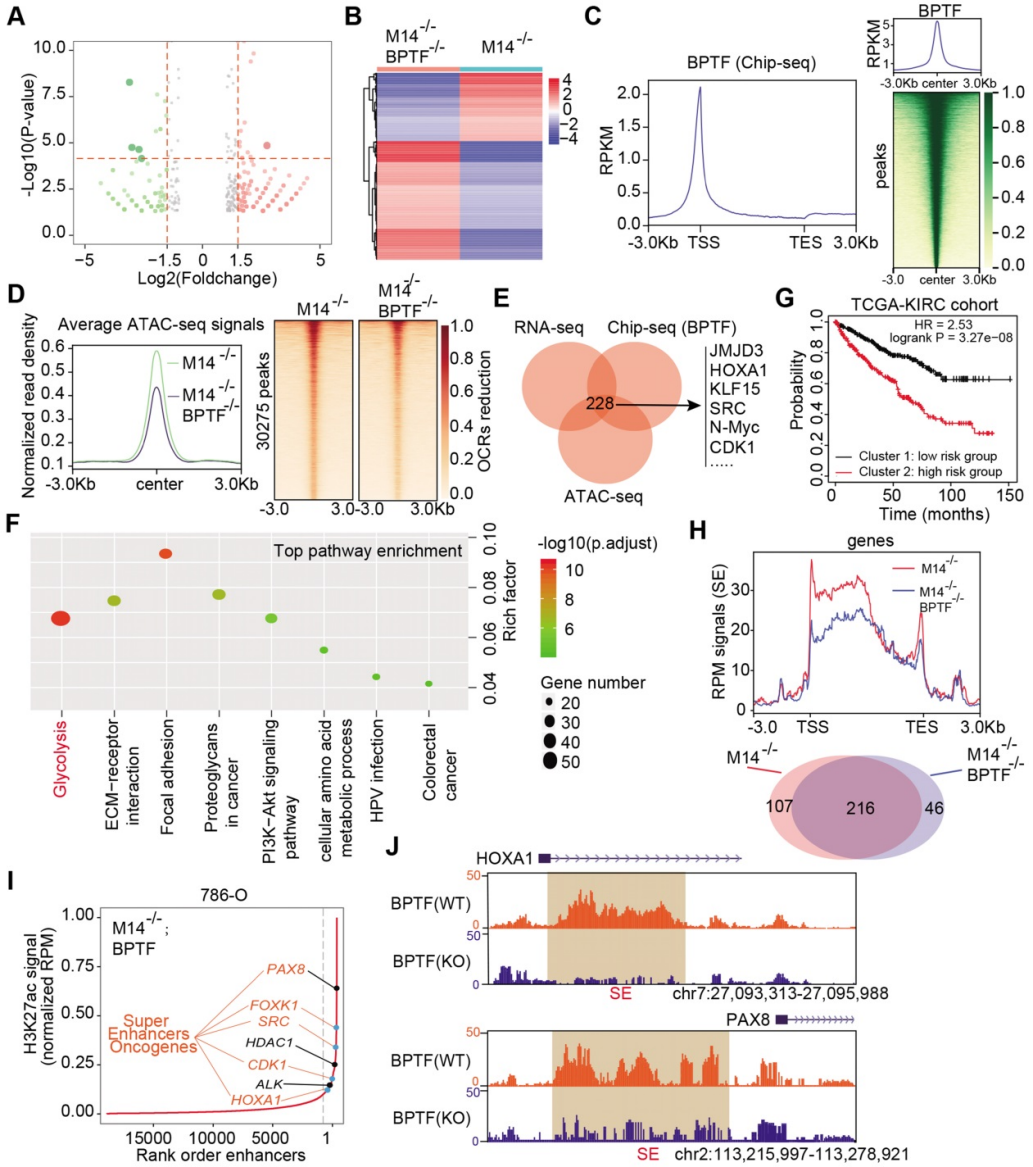
** Accumulated BPTF drives the oncogenic transcriptome and remodels the enhancer landscape in METTL14^-/-^ cells. (A-B)** Volcano plot and heatmap summarizing the DEGs in METTL14^-/-^ cells with and without BPTF ablation. **(C)** Left: metagene analysis of the genome-wide distribution and profile of BPTF. Right: heatmap summarizing the BPTF ChIP-seq signals -3 kb to +3 kb around the center of each peak. **(D)** Left: ATAC-seq analysis of the OCRs profiles across the genome-scale peaks in METTL14^-/-^ cells with and without BPTF ablation. Right: heatmap summarizing the ATAC signals -3 kb to +3 kb around the center of each peak with 30,275 peaks detected to have reduced signals upon BPTF loss. **(E)** BPTF signature identified by overlapping ChIP-seq, RNA-seq, and ATAC-seq in METTL14^-/-^ cells. **(F)** KEGG pathway analysis of the BPTF signature in METTL14^-/- or low^ cells. **(G)** OS (left) and metastatic cases (right) of patients in the TCGA-KIRC cohort stratified by BPTF signature using an unsupervised clustering algorithm. **(H)** Above: BPTF-related SEs signals across genomic H3K27ac peaks in METTL14^-/-^ cells. Below: Venn diagram identifying 46 specific BPTF-related SEs in METTL14^-/-^ cells. **(I-J)** Representative BPTF-related SEs in METTL14^-/-^ cells and specific putative SEs peaks across indicated gene loci identified using the ROSE algorithm.

To explore the downstream mechanisms mediated by the METTL14/BPTF axis in metastatic RCC, we conducted RNA-seq with METTL14^-/-^ cells and double-knockout (METTL14^-/-^, BPTF^-/-^) cells. Differential analysis was performed to identify 440 altered genes with |fold change| > 2 and *P* < 0.05, of which 279 were upregulated and 161 were downregulated in the volcano plot (Figure 5A-B). In addition, to determine the putative downstream targets of BPTF, we conducted ChIP-seq using the anti-BPTF antibody in METTL14^-/-^ cells. The average plot and ChIP-seq heatmaps confirmed that BPTF mainly distributed across promoters and intergenic regions (Figure 5C, Figure S4A-B). Considering that BPTF constitutes a subunit of the NURF complex (ATP-dependent remodeling factor) that mediates nucleosome movement and alters chromatin structures, we reasoned that BPTF ablation may cause aberrant chromatin configurations in METTL14^-/-^ cells. To test this speculation, ATAC-Seq was conducted with METTL14^-/-^ and double-knockout (METTL14^-/-^, BPTF^-/-^) 786-O cells, and the differential open chromatin regions (OCRs) were compared. The genome-wide ATAC-seq signals showed that BPTF ablation resulted in a nearly 20% reduction in OCRs compared with the effect of BPTF ablation in intact cells (Figure 5D). Specifically, 30,275 peaks were found to exhibit reduced DNA accessibility in the double-knockout (METTL14^-/-^, BPTF^-/-^) cells compared to the control cells (Figure 5D). Considering these results, we recognized that BPTF loss can contribute to notable changes in chromatin dynamics in METTL14^-/- or low^ cells. Accordingly, we overlapped the ATAC-seq, RNA-seq, and ChIP-seq data and identified 228 putative targets of BPTF, termed the BPTF signature (Figure 5E, Table S3). KEGG pathway analysis indicated that the BPTF signature was mainly enriched in tumor-related pathways, of which the top hit was the glycolysis pathway (Figure 5F). Utilizing the unsupervised clustering algorithm based on this BPTF signature, we successfully classified TCGA-KIRC patients into high- and low-risk groups that exhibited differential survival outcomes (Figure 5G). Intriguingly, the Chi-squared test showed that cluster 2 contained significantly more metastatic KIRC-RCC cases than cluster 1, supporting the associations between the BPTF signature and RCC metastasis (Figure S4C).

BPTF is known to interact with MYC, which recruits various transcription factors to induce transcriptional activation. Considering that BPTF binds mainly to distal enhancer elements, we thus reasoned that accumulated BPTF may constitute and reinforce enhancers or super-enhancers (SEs) to activate downstream targets. In addition, the genome-wide profiles of BPTF-related enhancers in RCC have never been illustrated or reported. We performed ChIP-seq with anti-H3K27ac, an enhancer indicator, to compare the differential enhancer landscape in METTL14^-/-^ versus double-knockout (METTL14^-/-^, BPTF^-/-^) 786-O cells. As shown in Figure 5H, enrichment of H3K27ac signals near the transcription start site (±1 kb) and enhancer regions were reduced upon BPTF knockout relative to control cells. In particular, we focused on the screening of SEs and produced an SE ranking plot based on H3K27ac signals using the rank ordering of super-enhancers (ROSE) algorithm with two conditions. Relative to the control cells, a series of 46 putative BPTF-related SE regions were discovered, and they were specifically reduced upon BPTF ablation (Table S3). Among these putative H3K27ac peaks, we identified several well-known SE-mapped oncogenic genes that are also included in the BPTF signature, such as PAX8, SRC, FOXK1, CDK1, and HDAC1 (Figure 5I). The representative SE tracks within the putative regions of several genes are illustrated in Figure 5J. Thus, we conclude that accumulated BPTF may remodel the enhancer landscape to alter a series of oncogenic signatures in METTL14^-/-^ RCC cells.

### BPTF constitutes SEs that activate ENO2 and SRC in METTL14^low^ cells, resulting in glycolytic reprogramming

**Figure 6:**
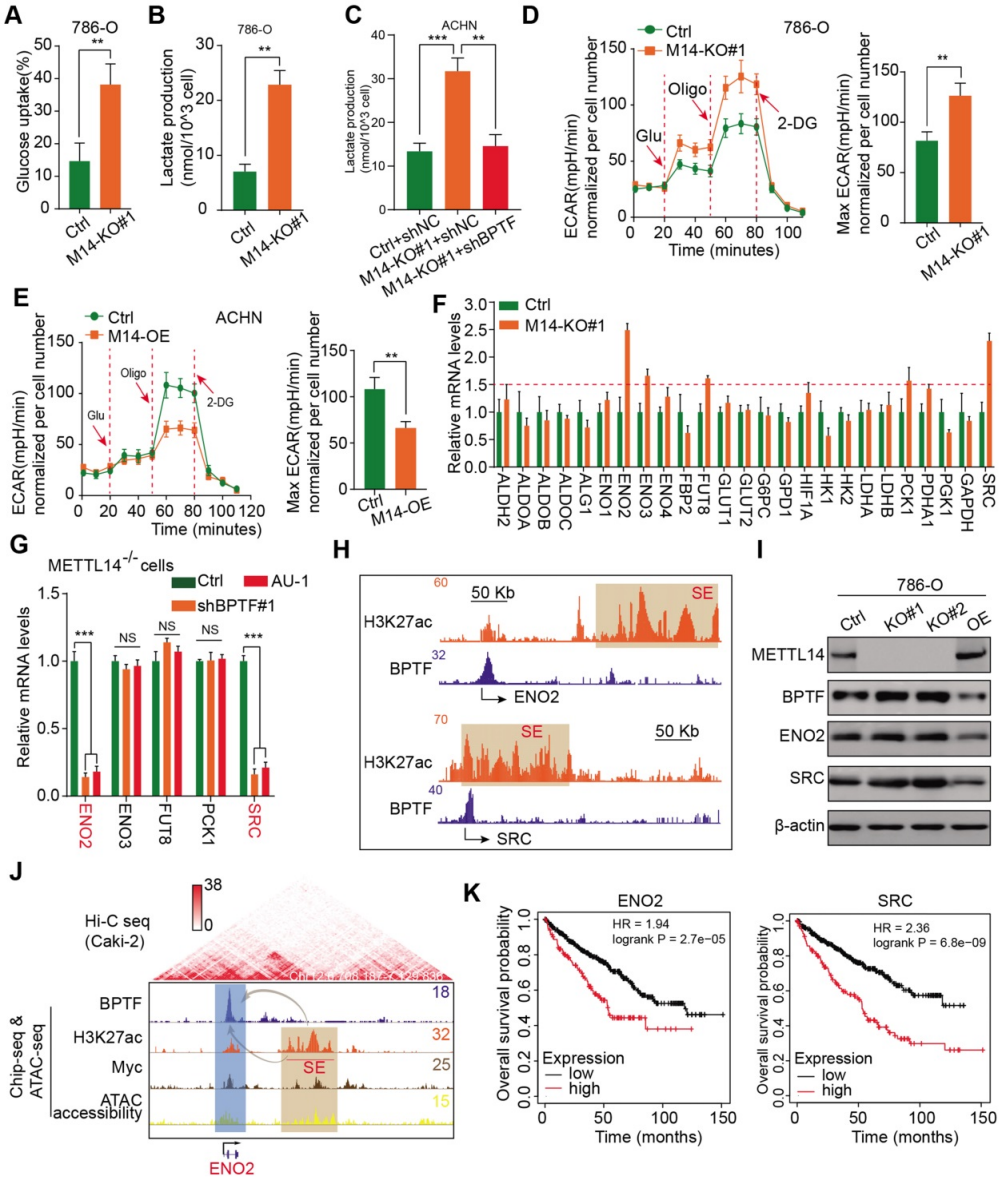
** METTL14/BPTF crosstalk mainly drives glycolytic reprogramming by activating ENO2 and SRC via SEs. (A-B)** Glucose uptake (A) and lactate production (B) in WT and METTL14^-/-^ (M14-KO#1) 786-O cells. **(C)** BPTF-dependent lactate production in METTL14^-/-^ ACHN cells. **(D-E)** Extracellular acidification rate profiles in WT, METTL14^-/-^, and METTL14-overexpressing (M14-OE) 786-O cells detected by Seahorse XF24. The specific inhibitors are provided at indicated points. **(F)** mRNA alterations in glucose metabolism-associated genes and BPTF signature upon METTL14 knockout in 786-O cells. **(G)** mRNA alterations upon BPTF knockdown or inhibition in METTL14^-/-^ 786-O cells. **(H)** BPTF peaks and SEs (indicated by H3K27ac peaks) across the ENO2 and SRC putative loci. **(I)** Western blots of the METTL14/BPTF axis, ENO2, and SRC in 786-O cells. **(J)** ChIP-seq, ATAC-seq, and public Hi-C data illustrating the proximity of the BPTF-directed SEs near ENO2 and their co-appearance with MYC transcription, enabling the prediction of 3D structural R-looping that brings SEs to the ENO2 promoter. **(K)** Kaplan-Meier analysis of OS stratified by ENO2 (left) and SRC (right) expression in the TCGA-KIRC cohort.

Extensive studies have demonstrated that the aerobic glycolytic phenotype of RCC is often correlated with tumor progression, angiopoiesis, and metastasis. Considering that the top hit among enriched pathways in the BPTF signature was aberrant glycolysis metabolism, as shown in Figure 5F, we sought to confirm that the METTL14/BPTF axis regulates the RCC glycolytic process. METTL14 deficiency significantly enhanced glucose uptake and lactate production compared with that of the control cells (Figure 6A-B). Additionally, BPTF knockdown reduced lactate production in METTL14^-/-^ cells (Figure 6C). The kinetic profiles, including the extracellular acidification rate, demonstrated that glycolytic activity notably increased upon METTL14 loss and was reduced with forced expression of METTL14 (Figure 6D-E). To comprehensively screen the specific glycolytic targets of METTL14/BPTF crosstalk in RCC, we searched a series of glucose metabolism-associated genes and BPTF signature and observed that ENO2, ENO3, FUT8, PCK1, and SRC were notably enhanced in METTL14^-/-^ cells, but only ENO2 and SRC were consistently reduced upon BPTF knockdown or treatment with the BPTF inhibitor AU1 (Figure 6F-G, Figure S4D). Additionally, Integrated Genomics Viewer diagrams verified the co-occurrence of SEs and BPTF-binding peaks near the same ENO2 and SRC regions (Figure 6H). Along with BPTF, ENO2 and SRC accumulated upon METTL14 ablation and decreased with ectopic expression of METTL14, as determined by western blot assays (Figure 6I). We combined ChIP-seq, ATAC-seq, and high-throughput chromosome conformation capture (Hi-C) data obtained from the ENCODE dataset (https://www.encodeproject.org/) to further confirm the direct R-loop interactions in RCC cells from several perspectives. These potential loops enable the physical interactions of SEs, BPTF, and c-Myc with the promoter of ENO2 to facilitate transcription (Figure 6J). Moreover, Kaplan-Meier analysis of the TCGA-KIRC cohort further demonstrated that high levels of ENO2 and SRC were associated with poor OS outcomes (log-rank test, *P* < 0.001, Figure 6K). Overall, these data indicate that the METTL14/BPTF axis can partly drive RCC metastasis by enhancing the glycolytic process.

### BPTF antagonist, AU1, inhibits METTL14^-/- or low^ RCC tumor metastasis

**Figure 7:**
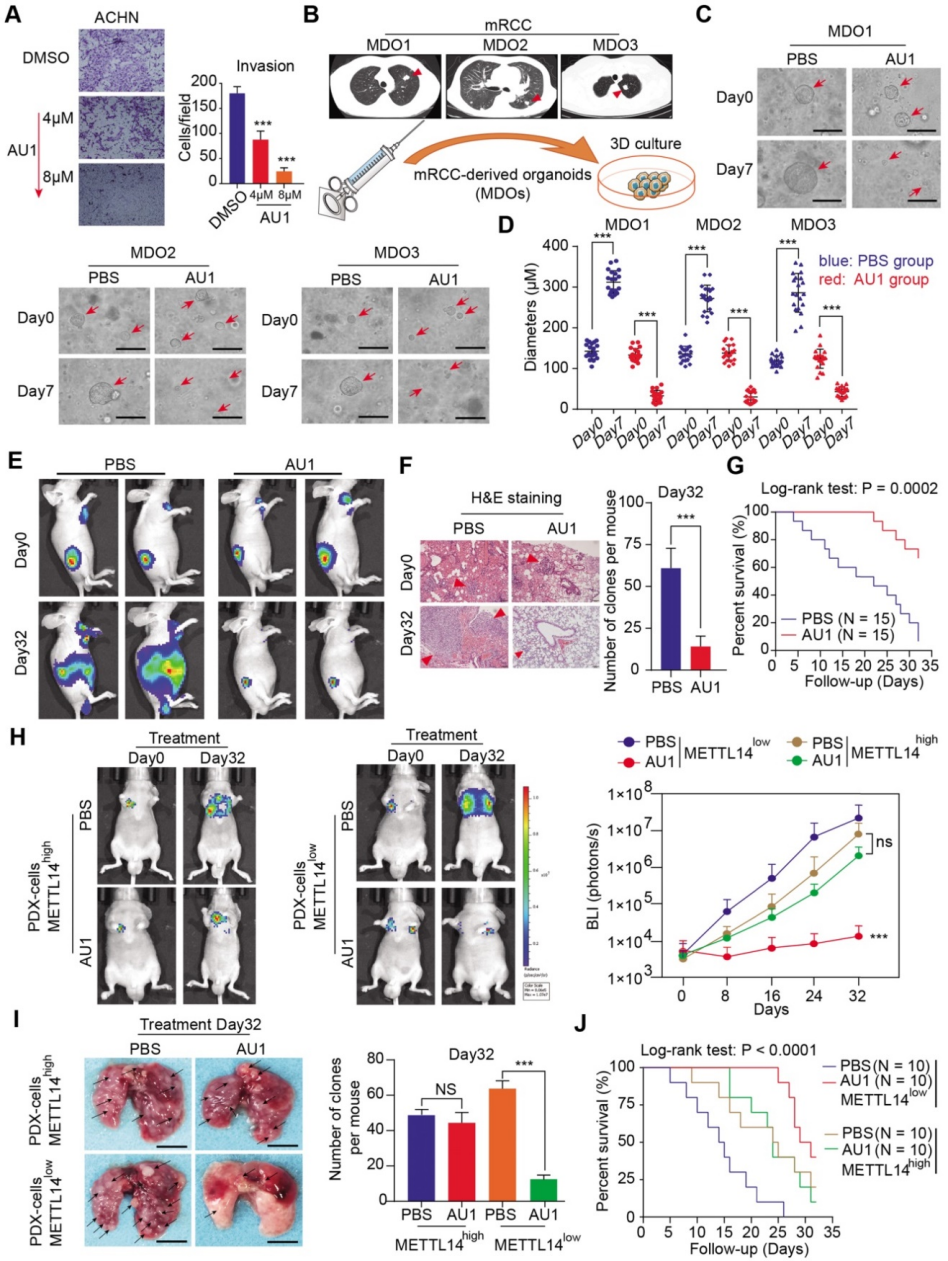
** Assessment of the clinical significance of the BPTF inhibitor AU1 in suppressing METTL14^-/- or low^ RCC metastasis. (A)** AU1 dose-dependent inhibition of the invasion abilities of ACHN cells. **(B)** CT-guided needle biopsies of lung metastases from three eligible mRCC patients for establishment of MDO models. **(C-D)** Wide field microscopy images and size quantitation of the three MDO models treated with AU1. Scale bar = 300μM **(E-G)** *In vivo* BLI, H&E staining, and survival following AU1 treatment of an orthotopic xenograft model generated with METTL14^-/-^ Luc-Renca cells. **(H-I)** *In vivo* BLI, white light images of excised lungs, and Kaplan-Meier analysis following AU1 treatment of metastatic models generated with METTL14^low^ and METTL14^high^ patient-derived cells.

**Figure 8:**
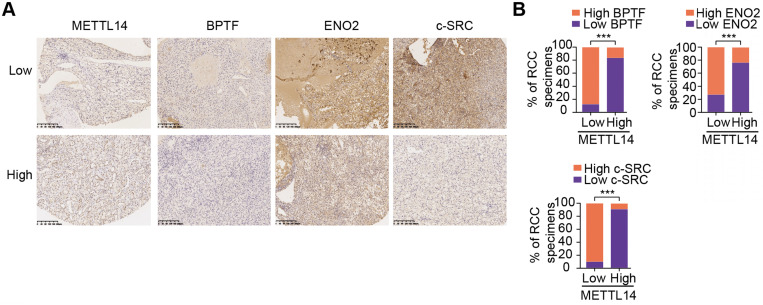
** Clinical validation of METTL14/BPTF and glycolytic crosstalk in RCC. (A)** Representative IHC staining and **(B)** quantitation of BPTF, ENO2, and SRC expressions in 70 RCC specimens stratified by METTL14 expression.

Given the important mechanisms of the METTL14/BPTF axis in driving RCC metastasis, we sought to confirm the clinical significance of BPTF inhibitors in restraining acquisition of a metastatic phenotype. First, we detected the half maximal inhibitory concentration of the BPTF antagonist AU1 and found that it significantly suppressed RCC cell migration and invasion *in vitro* (Figure 7A, Figure S5A). Currently, patient-derived organoids are considered to exhibit a high degree of similarity to original tumors and are useful preclinical models 28-30. To establish MDO models, we considered a total of 10 patients with metastatic RCC treated between January 2017 and December 2019 at Ruijin Hospital, and selected 3 CT-guided needle lung biopsies with low expression of METTL14 (H-scores < median 18.2) (Figure 7B). We accordingly established MDO models and found that AU1 significantly restrained organoid size compared with PBS treatment in a time- and dose-dependent manner (Figure 7C-D, Figure S5B). To further demonstrate the active roles of the METTL14/BPTF axis in RCC metastasis *in vivo*, we employed an orthotopic xenograft model generated using METTL14^-/-^ Luc-Renca cells. These modified Renca cells were implanted into the subrenal capsule of the right kidney of BALB/c mice. We monitored metastasis into the lung by BLI and enrolled 30 mice with relatively equal degrees of metastasis into two groups (N = 15 each) for subsequent drug trials. The enrolled mice were treated i.p. with either AU1 (13.2 mg/kg) or PBS three times per week for 5 weeks. We found that AU1 effectively inhibited distal lung metastases compared to control mice treated with PBS, as quantified by BLI and the number and size of the lung metastases (Figure 7E-F, Figure S5C). In addition, mice in the control group had significantly worse prognosis than those administered with AU1 (log-rank test, *P* = 0.0002, Figure 7G). Considering that tumor heterogeneity is a determinant of cancer progression and evolution, we questioned whether distinct METTL14 expression levels can influence the drug efficiency of AU1 in mRCC. We thus derived two patient-derived RCC cells with high and low METTL14 expression levels, as validated by IHC results (Figure S5D). The *in vivo* models suggested that AU1 can notably suppress the metastatic abilities of METTL14^low^ cells but exerted marginal effects on METTL14^high^ cells, as quantified by serial BLI and the number and size of the lung metastases (Figure 7H-I). Kaplan-Meier analysis further suggested that AU1 can significantly improve the prognosis of mice injected with METTL14^low^ cells but not those injected with METTL14^high^ cells (Figure 7J). Finally, to confirm the robustness of the METTL14/BPTF axis in enhancing the RCC glycolysis process, we further selected patients with distinct METTL14 expression levels and assessed the relevance of this axis. In line with previous results, the IHC data suggested that METTL14 correlated negatively with the expression levels of BPTF, ENO2, and SRC in 70 patients from the Ruijin-RCC dataset (Figure 8A-B).

## Discussion

Among the common types of modifications to mRNA or noncoding RNA, m^6^A is the current hot topic as it is known to play essential roles in physiological and pathological processes, including tumorigenesis 31-33. Despite extensive knowledge of m^6^A modification enzymes in various cancers, their roles in renal cell carcinoma and RCC metastasis have been less reported. Using high-throughput sequencing data of RCC samples, we found that METTL14 expression is low in metastases and possesses independent prognostic significance in RCC. Distant metastasis is recognized to be the most hazardous feature in RCC, leading to rapid tumor progression and poor response to drugs 34, 35.

BPTF, the largest subunit of the NURF chromatin complex, mediates ATP-dependent dynamic nucleosome sliding and DNA accessibility. Previous studies have reported that BPTF is an essential epigenetic regulator in various cancers, but little is known about its effect in RCC, especially with respect to metastasis. As the direct downstream gene of METTL14, BPTF mainly regulates glycolytic reprogramming in RCC. By detecting a panel of glucose metabolism-related genes in the BPTF signature, we ultimately identified ENO2 and SRC as the potential targets of the METTL14/BPTF axis. Accumulated BPTF in METTL14-deficient cells altered the enhancer landscape and reinforced SEs to activate ENO2 or SRC. ENO2 is a cytoplasmic glycolytic enzyme that can function as a novel metabolic biomarker for predicting pancreatic ductal adenocarcinoma metastasis and as a metabolic therapeutic target 35. In addition, SRC is known to promote tumor progression and glycolysis by regulating PFKFB3 activity 36-38. Compared to typical enhancers, SEs could regulate wider genomic ranges and possess several folds of activated efficiency 39. Targeting SEs has recently provided novel insights into cancer treatment, and inhibitors like JQ1 and HDAC were demonstrated to be effective 40-42. Given the essential roles of ENO2 and SRC in modulating glycolysis and the lack of available METTL14-related drugs, reduction of BPTF-mediated SEs may significantly inhibit the expressions of both, leading to suppression of glycolysis-driven metastasis. In addition, IHC results from a large group of RCC samples further supported the idea that METTL14 expression is positively associated with BPTF, ENO2, and SRC, suggesting the robustness of the METTL14/BPTF axis in enhancing RCC glycolysis. For the first time, we established MDOs to mimic the actual metastatic niche and assessed the suppressive activity of the BPTF inhibitor AU1. Limited by the eligibility of only three MDOs, we intend to collect more MDOs to determine AU1 efficiency in our subsequent studies. Notably, cell viability assays and patient-derived cell-based models revealed that AU1 was only effective in the METTL14^low^ but not the METTL14^high^ cases, implicating tumor heterogeneity across samples. We speculate that there might be a lack of AU1-druggable targets in METTL14^high^ RCC cells, contributing to the limited responses.

Aerobic glycolysis, known as the Warburg effect, is regarded as a prominent hallmark in ccRCC and is characterized by increased glucose uptake and lactate production even under normal oxygen conditions 43, 44. Aerobic glycolysis efficiently produces the energetic and biosynthetic requirements for the sustained growth and metastasis of cancer cells 45-47. Additionally, glycolysis can also affect secretion of immune factors into the microenvironment and promote tumor angiogenesis, which leads to distant metastasis 48. Targeting direct glucose transporters and glycolytic enzymes or inhibiting glycolysis-related mediators has been demonstrated to be a powerful therapeutic strategy for suppressing the proliferation and metastasis of tumors 45, 49. In the current study, BPTF was demonstrated to activate ENO2 and SRC, serving as an efficacious switch to manipulate RCC glycolysis. Although glycolysis reprogramming was a top hit in our high-throughput screening, whether other mechanisms underlie METTL14/BPTF axis action is an intriguing idea to explore further.

Nevertheless, there are several limitations to our study. First, we utilized IHC scores to classify the METTL14^low^ and METTL14^high^ samples. However, how to define the optimal METTL14 cutoff for drug administration is an essential issue to determine. In addition, more rigorous preclinical models are warranted to comprehensively evaluate the specific efficacy and drug safety of AU1 in mRCC. In addition, whether METTL14 leads to the acquisition of other phenotypes, such as tumor immunity, stemness traits, or apoptosis in RCC is worth systematically elucidating.

Taken together, our study describes the essential roles of METTL14-mediated m^6^A modification in mRCC. The METTL14/BPTF axis was demonstrated to drive glycolytic reprogramming and metastasis partly through BPTF-constituting SEs at ENO2 and SRC. The clinical BPTF inhibitor AU1 was effective in suppressing distal metastases in METTL14^-/-or low^ RCC models.

## Supplementary Material

Supplementary figures and table S1-2.

Supplementary table S3.

## Abbreviations

ATAC: assay for transposase-accessible chromatin
BLI: bioluminescence imaging
BPTF: bromodomain PHD finger transcription factor
ChIP: chromatin immunoprecipitation
DEGs: differentially expressed genes
EMT: epithelial-mesenchymal transition
ENO2: enolase 2
GO: Gene Ontology
Hi-C: high-throughput chromosome conformation capture
ICGC: International Cancer Genome Consortium
IHC: immunohistochemistry
KIRC: kidney renal clear cell carcinoma
KO: knockout
MDOs: metastatic renal cell carcinoma patient-derived organoids
MeRIP-seq: methylated RNA immunoprecipitation sequencing
METTL14: methyltransferase-like 14
mRCC: metastatic renal cell carcinoma
NURF: nucleosome remodeling factor
OCRs: open chromatin regions
OS: overall survival
PFS: progression-free survival
qRT-PCR: quantitative real-time polymerase chain reaction
RCC: renal cell carcinoma
RIP: RNA immunoprecipitation
ROSE: rank ordering of super-enhancers
SE: super-enhancer
siRNA: small interfering RNA
SRC: SRC proto-oncogene nonreceptor tyrosine kinase
TCGA: the cancer genome atlas
WT: wildtype
