# Supplementary material for: Downregulated METTL14 accumulates BPTF that reinforces super-enhancers and distal lung metastasis via glycolytic reprogramming in renal cell carcinoma
Source: Theranostics. 2021 Jan 26;11(8):3676–93. doi: 10.7150/thno.55424 (PMC7914369; doi:10.7150/thno.55424)
Supplement: Supplementary file 1 — Supplementary figures and table S1-2. [file thnov11p3676s1.pdf]

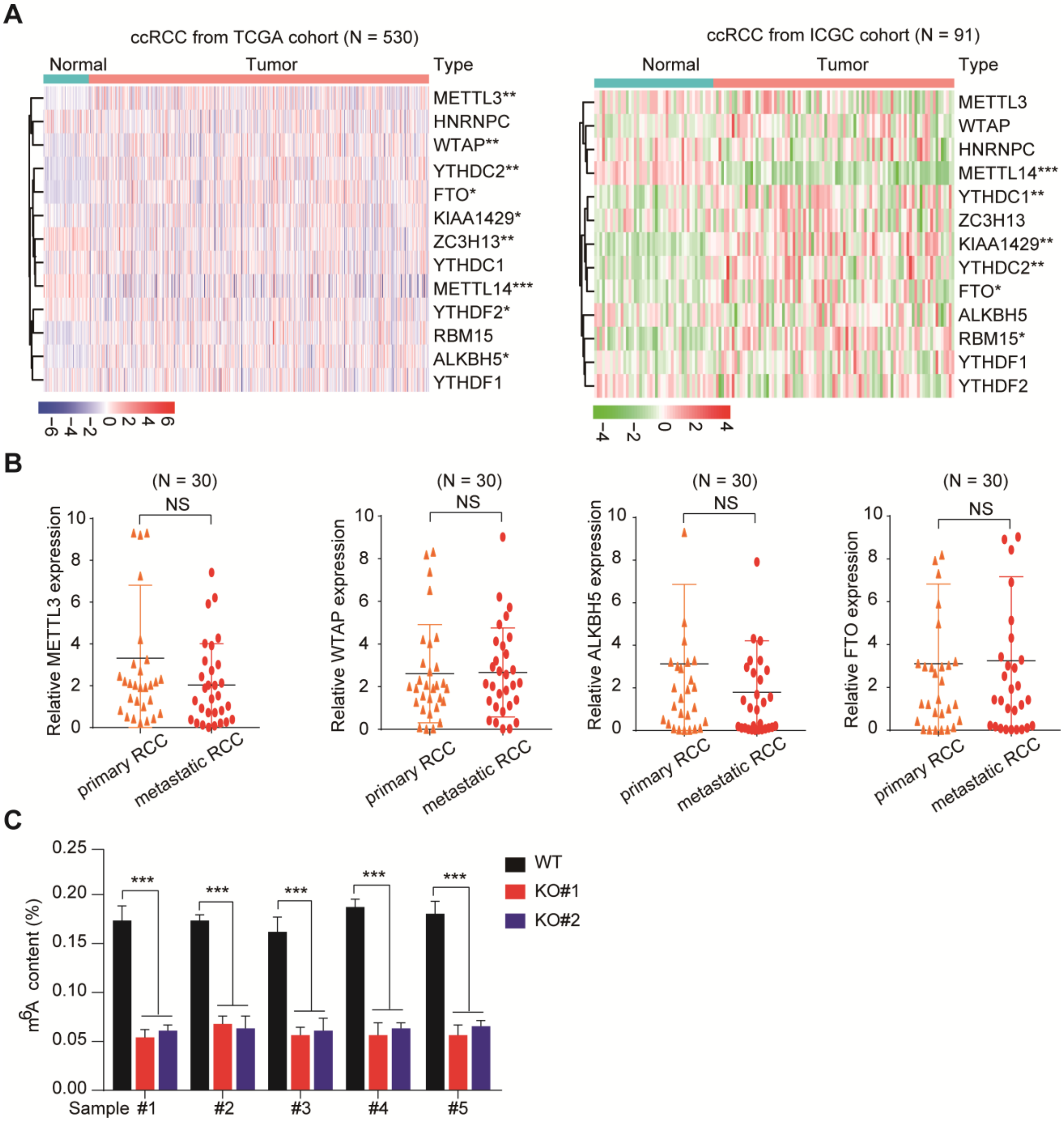

Fig. S1 METTL14 expressed lowly in TCGA-KIRC cohort and Ruijin-RCC samples compared with other known m^{6}A enzymes. (A) The differential expression levels of 13 well-known m^{6}A-related enzymes were calculated and compared between normal and tumor tissues, in which METTL14 was notably observed to down-regulate in RCC tumor samples in both TCGA- and ICGC-RCC cohorts. (B) Besides, we obtained 30 paired primary- and metastatic-RCC samples and detected that METTL14 was the only one to express lowly in mRCC tissues relative to other enzymes, like METTL3, WTAP, ALKBH5 and FTO. (C) The indicated METTL14-knockout cells were used for m^{6}A detection.

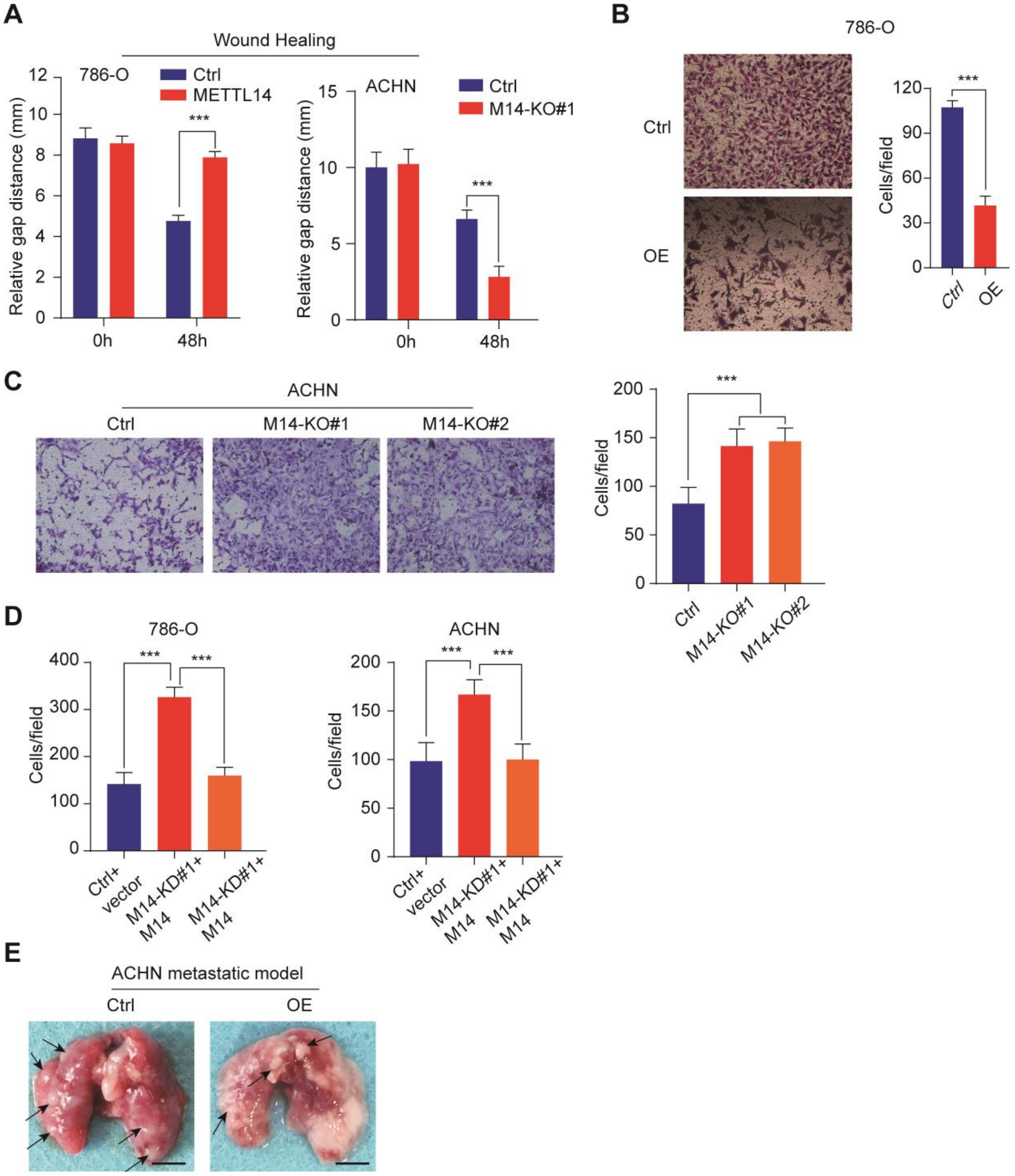

Fig. S2 METTL14 correlated with metastasis *in vitro* and *in vivo*. (**A)** METTL14 overexpression significantly impeded the invasive ability relative to ctrl cells via wound-healing assay in 786-O (left), whereas METTL14 deficiency promoted the invasive ability of ACHN, indicated by relative gap distance (right). (**B)** Meanwhile, METTL14 overexpression also suppressed the migration ability in 786-O compared with control cells. (**C)** Additionally, METTL14 deficiency enhanced the invasive ability of ACHN, indicated by transwell assay, indicated by cells/fields. (**D)** Detection of migratory and invasive abilities of RCC cells in over-expressed METTL14 in METTL14-knockout cells and control cells. **(E)** The tail vein metastasis model

further showed that the numbers of lung metastases were notably fewer in METTL14-OE groups than those in control groups.

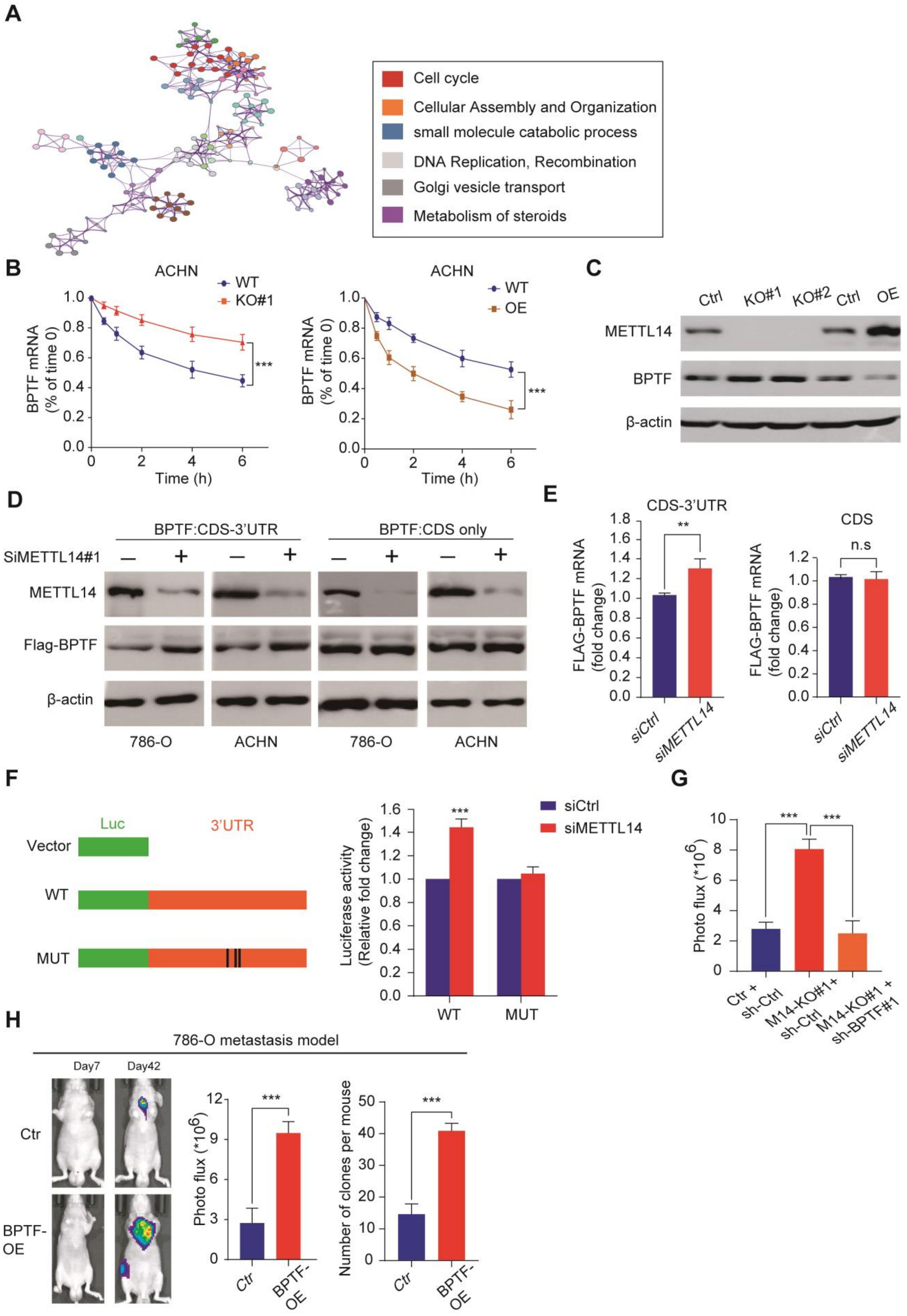

A
Cell cycle
Cellular Assembly and Organization
small molecule catabolic process
DNA Replication, Recombination
Golgi vesicle transport
Metabolism of steroids
B
ACHN
WT
KO#1
BPTF mRNA (% of time 0)
Time (h)
***
ACHN
WT
OE
***
C
Ctrl
KO#1
KO#2
Ctrl
OE
METTL14
BPTF
β-actin
D
BPTF:CDS-3'UTR
BPTF:CDS only
SiMETTL14#1
METTL14
Flag-BPTF
β-actin
786-O
ACHN
786-O
ACHN
E
CDS-3'UTR
**
FLAG-BPTF mRNA (fold change)
siCtrl
siMETTL14
CDS
n.s
F
Luc
3'UTR
Vector
WT
MUT
Luciferase activity (Relative fold change)
***
siCtrl
siMETTL14
WT
MUT
G
***
***
Photo flux (*10^6)
Ctr + sh-Ctrl
M14-KO#1+ sh-Ctrl
M14-KO#1 + sh-BPTF#1
H
786-O metastasis model
Day7
Day42
Ctr
BPTF-OE
***
Photo flux (*10^6)
Ctr
BPTF-OE
***
Number of clones per mouse
Ctr
BPTF-OE

Fig. S3 METTL14 correlated with oncogenic pathways and BPTF was the bona fide downstream target. **(**A) The Gene Ontology (GO) analysis based on the differential genes (DEGs) indicated that METTL14 correlated with oncogenic crosstalk, including cell cycle, cellular assembly and organization, small molecule catabolic process and DNA replication, recombination. (B) Upon the treatment of actinomycin D, METTL14 deficiency enhanced the BPTF mRNA stability, whereas METTL14 overexpression exhibited the opposite results in ACHN. (C) Detection of BPTF protein levels in METTL14 intact, METTL14 deficiency and METTL14 over-expression cells via western-blotting, matched to Figure 3M. (D-E) Upon METTL14 knockdown, FLAG-BPTF expression was significantly increased in RCC cell lines transfected with BPTF CDS-3′UTR construct, but not the BPTF CDS-alone construct, which was validated by western-blot (c) and qPCR (d). (F) The BPTF 3’UTR-reporter luciferase assay showed that targeting METTL14 via specific siRNAs significantly enhanced the activity of the luciferase of the BPTF-WT-3’UTR construct, whereas had no effect on the Mut-3’UTR construct with mutations at the unique peak sites. (G) The *in vivo* metastatic model revealing that BPTF knockdown significantly suppressed the tumor metastatic ability of METTL14$^{-/-}$ cells, as quantified by the luciferase signals. (H) The *in vivo* BPTF-OE effect on RCC metastasis was assessed compared with control group.

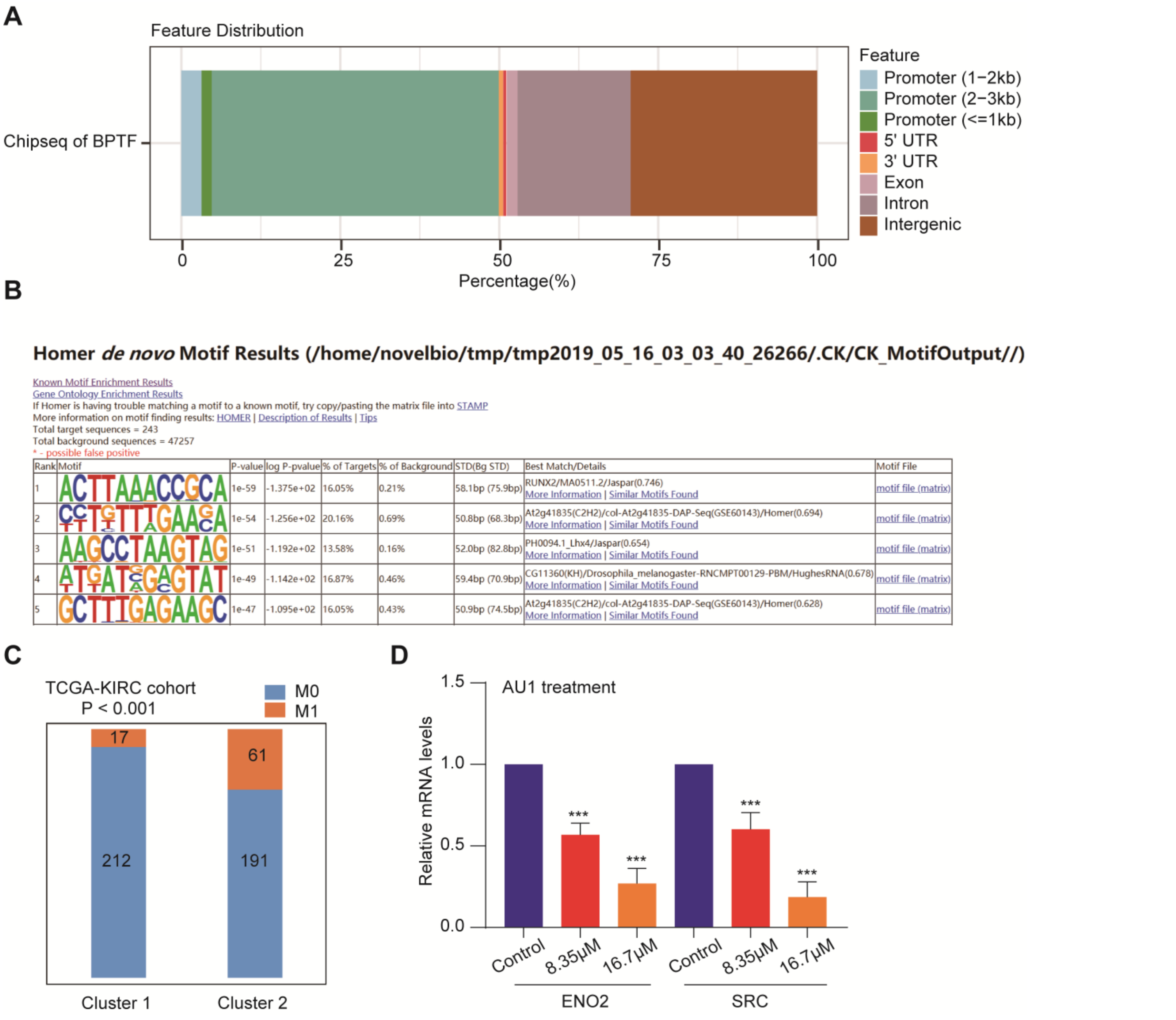

Homer *de novo* Motif Results (/home/novelbio/tmp/tmp2019_05_16_03_03_40_26266/.CK/CK_MotifOutput//)

Known Motif Enrichment Results
Gene Ontology Enrichment Results
If Homer is having trouble matching a motif to a known motif, try copy/pasting the matrix file into STAMP
More information on motif finding results: HOMER | Description of Results | Tips
Total target sequences = 243
Total background sequences = 47257
* - possible false positive

Rank	Motif	P-value	log P-pvalue	% of Targets	% of Background	STD(Bg STD)	Best Match/Details	Motif File
1	ACTTAAACCGCA	1e-59	-1.375e+02	16.05%	0.21%	58.1bp (75.9bp)	RUNX2/MA0511.2/Jaspar(0.746) More Information | Similar Motifs Found	motif file (matrix)
2	CCTGTTTGAAGA	1e-54	-1.256e+02	20.16%	0.69%	50.8bp (68.3bp)	At2g41835(C2H2)/col-At2g41835-DAP-Seq(GSE60143)/Homer(0.694) More Information | Similar Motifs Found	motif file (matrix)
3	AAGCCTAAGTAG	1e-51	-1.192e+02	13.58%	0.16%	52.0bp (82.8bp)	PH0094.1_Lhx4/Jaspar(0.654) More Information | Similar Motifs Found	motif file (matrix)
4	ATGATCGAGTAT	1e-49	-1.142e+02	16.87%	0.46%	59.4bp (70.9bp)	CG11360(KH)/Drosophila_melanogaster-RNCMPT00129-PBM/HughesRNA(0.678) More Information | Similar Motifs Found	motif file (matrix)
5	GCTTTGAGAAGC	1e-47	-1.095e+02	16.05%	0.43%	50.9bp (74.5bp)	At2g41835(C2H2)/col-At2g41835-DAP-Seq(GSE60143)/Homer(0.628) More Information | Similar Motifs Found	motif file (matrix)

Figure S4. Chip-seq data analysis of BPTF in RCC. (A-B) Illustration of BPTF distributions across the genomic range and the binding motif analysis. **(C)** The Chi-squared test was conducted to reveal that cluster 2 contained significantly more metastatic KIRC-RCC cases than cluster 1**.** (**D**) The detection of mRNA levels of ENO2 and SRC after different doses of AU1 treatment via qPCR.

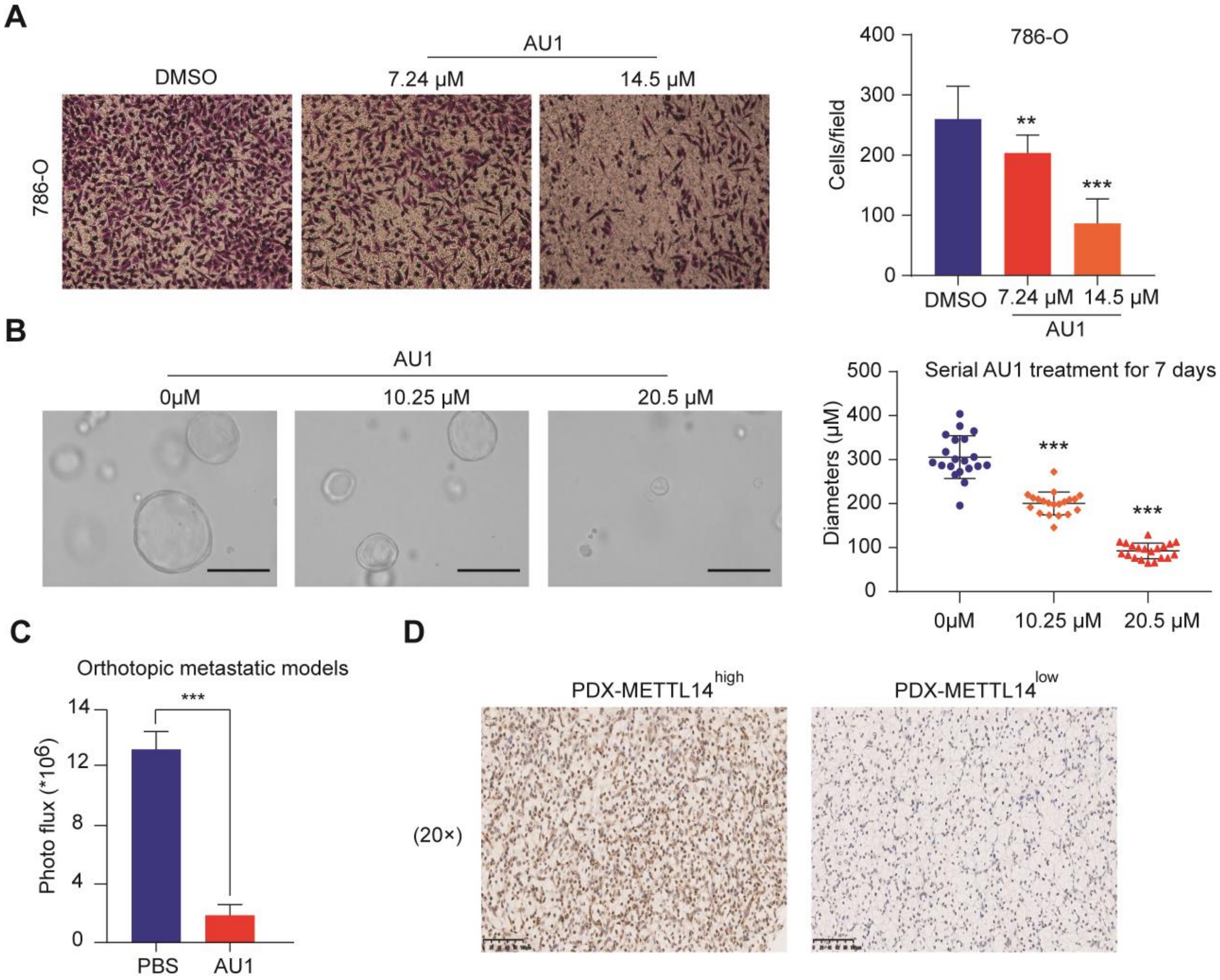

Figure S5. The BPTF inhibitor, AU1, could efficiently inhibit the metastatic abilities of METTL14-deficient RCC cells in vitro and in vivo. (A) AU1 could significantly inhibit the invasion of 786-O cells in a dose-dependent manner. (B) AU1 could significantly restrain the sizes of MDO compared with PBS treatment in a dose-dependent manner. Scale bar = 300μM (C) In the orthotopic model, AU1 could significantly inhibit the distal lung metastasis relative to PBS treatment, indicated by signals of bioluminescence imaging (BLI). (D) The PDX cells was obtained from two eligible RCC-PDX samples with distinct METTL14 expression levels, validated by IHC staining.

Table S1. Primers, sequences of shRNAs and siRNAs, antibody and chemicals.

Primers for RT-qPCR with cell lines samples		
Gene name	F: 5'-3'	R: 5'-3'
METTL14	GGCATGGACTGTGGTCATGAG	CTCATGACCACAGTCCATGCC
BPTF	TTACCAAGAGCAGCAAGAAGAGCATT	CATCACTTGGAGGTATAGACTTCAGACAG
GAPDH	TGCACCACCAACTGCTTAGC	GGCATGGACTGTGGTCATGAG
CDK1	GTCTTCAGGATGTGCTTATG	ATTCTATCCCTCCTGGTCA
KLF4	GTCTTGAGGAAGTGCTGAG	CCATTACCAAGGTCAGTCC
CHD1	CAATCATCGTCTCCTTATCAC	CTGTTACGCCGAGTTAAGA
C3orf20	CTCCATCACAGTCACCTTC	AAGGTGTATAAGATGAGCCG
MYLK3	CTGGATGTGGTCCTGTTC	TCAATCAGACAGGACATCAA
NR1D1	CATCCTCCTCCTCCTTCTAT	CAAGAGCACCAGCAACAT
FOXE1	GCGGCATCTACAAGTTCA	CTCAACGACTGCTTCCTC
CDC20	ATCCAGTTCCAAGGTTCAG	TTACCTGAACCTTGTGGATT
PROX1	AGAGTTGACATTGGAGTGAA	AGGTTCTGAGCAGGATGT
RUNX1	ATGGCTGGCAATGATGAA	TCCATTGCCTCTCCTTCT
SOX4	AAGACAGCGACAAGATCC	GGAAGAAGGTGAAGTCCG
PHGDH	CCTTACCAGTGCCTTCTC	GGATGTGAACTTGGTGAAC
BMP2	GGTGGAATGACTGGATTGT	ACAGAACTCAGTGCTATCTC
ALDH2	CGTGGTTGTGATGAAGGT	GATAGCAAGACCGAGCAG
ALDOA	GCAGGAGGAGTATGTCAAG	CCTCTTCGTCTCTAACCAC
ALDOB	CTGGCTGCTGTCTACAAG	TGAGTGAAGAGGATGCCA
ALDOC	TTCAACCTCAATGCCATCA	TGAAGGCAGTGGAGAAGA
ALG1	CAATGCTATGCGAGAAGAC	AGTATTATAGCCGCCTCATC
ENO1	CGAGATGGATGGAACAGAA	TCTGAAGTCATCCTGCCA
ENO2	TGTGGTGGAGCAAGAGAA	TTCTCTTGCTCCACCACA

ENO3	GAAGGATGCCACCAATGT	AGCATCTGAGTTCTATCGC
ENO4	ACCATTAACTGTGACTCCAT	GACAGCATCTATCACGCA
FBP2	GCTGCTGAACTCAATGCT	AAGAGAATAAGGACGCCATC
FUT8	CTATGCTACTGGTGGATGG	AGAAGAAGCCACCAAGAAG
GLUT1	GCAGGAGATGAAGGAAGAG	CTGTCTTCTATTACTCCACGA
GLUT2	CTGCTGTCTCTGTATTCCTT	TTGGATGAGTTATGTGAGCA
G6PC	TGGAGGAAGGAATGAATGTT	ACTACAGCAACACTTCCG
GPD1	AGGTGGCTGATGAGAAGT	CTATACAGCATCCTCCAGC
HIF1A	CTTCTGGATGCTGGTGATT	ACCAACAGTAACCAACCTC
HK1	CAACAGCCACAGTCAAGA	ATGATGACCTGTGGCTATG
HK2	TCTGCTTGCCTACTTCTTC	AGAAGGTGGAGATGGAGAA
LDHA	GAGAGTGCTTATGAGGTGAT	AATGGAATCTCAGACCTTGT
LDHB	AACTTGCTCTTGTGGATGT	GAGTCCGTCAGCAAGAAG
PCK1	GGCTGAAGAAGTATGACAAC	TGGATGTCAGAGGAGGATT
PDHA1	ATTGATGTGGAAGTGAGGAA	CACATCTACTCCAGCGAC
PGK1	GATTATTGGTGGTGGAATGG	CAAGAAGTATGCTGAGGCT
SRC	CAGGCTGAGGAGTGGTAT	ACTACAAGATCCGCAAGC
**Primers for RT-qPCR with FFPE RCC patient tumor samples with FFPE patient tumor samples**		
METTL14	GGCATGGACTGTGGTCATGAG	CTCATGACCACAGTCCATGCC
BPTF	TTACCAAGAGCAGCAAGAAGAGCATT	CATCACTTGGAGGTATAGACTTCAGA CAG
GAPDH	TGCACCACCAACTGCTTAGC	GGCATGGACTGTGGTCATGAG
METTL3	GTTGCTACACCACCTCTC	CAAGAAGGTCAGTCAGGAG
WTAP	GCAACACAACCGAAGATG	TTCAGAATGGCTTGGACTC
ALKBH5	GTTCCAGTTCAAGCCTATTC	CTCTGAGAACTACTGGCG
FTO	CTCGCATCCTCATTGGTAA	TACAACGGACAAGATGAAGT
**Sequences of sgRNAs for METTL14/BPTF**		

METTL14#1	GCTGAAAGTGCCGACAGCAT	
METTL14#2	GAACATGGATAGCCGCTTGC	
BPTF#1	GTTCCGCAGTACCTCGTAAA	
BPTF#1	ACCATTAGCTGCCGCCAGAA	

Sequences of shRNAs

Gene name	Sequence
BPTF#1	CCGGCCAGTAGAGAACAAGATCAAACTCGAGTTTGATCTTGTTCTCTACTGGTTTTTG
BPTF#2	CCGGGCGGCAGCTAATGAAGAAATTCTCGAGAATTTCTTCATTAGCTGCCGCTTTTTG
METTL14#1	CCGGGCTTACAAATAGCAACTACAACTCGAGTTGTAGTTGCTATTTGTAAGCTTTTT
METTL14#2	CCGGCCATGTACTTACAAGCCGATACTCGAGTATCGGCTTGTAAGTACATGGTTTTT

Sequences of siRNAs

Gene name	Sequence
BPTF#1	GCAGTATACCATTTGGAAT
BPTF#2	GCGTCTACCACAATCAATA
METTL14#1	GCATTGGTGCCGTGTTAAA
METTL14#2	GGATGAACTAGAAATGCAA

Antibody & Chemicals

**Name**	**Species**	**Cat. No**	**Source**
METTL14	Mouse	ab220031	abcam
BPTF	Rabbit	ab72036	abcam
Anti-Actin	Rabbit	AC028	abclonal
E-cadherin	Mouse	CST#14472	Cell Signaling Technology (CST)
N-cadherin	Rabbit	CST#13116	Cell Signaling Technology (CST)
Vimentin	Rabbit	CST#5741	Cell Signaling Technology (CST)
SRC	Rabbit	CST#2109	Cell Signaling Technology (CST)
ENO2	Rabbit	CST#9536	Cell Signaling Technology (CST)
Anti-FLAG	Mouse	M185-7	MBL
puromycin		S7417	Selleckchem

Actinomycin D		S8964	Selleckchem
**Organoids culture**			
**Additive**	**Source**	**Cat. No.**	**Concentration**
EGF	PeproTech	AF-100-15	50 ng/ml
Noggin	PeproTech	250-38	100 ng/ml
R-Spondin 1	PeproTech	120-38	500 ng/ml
Gastrin	Sigma-Aldrich	G9145	10 nM
FGF-10	PeproTech	100-26	10 ng/ml
FGF-basic	PeproTech	100-18B	10 ng/ml
Wnt-3A	R&D Systems	5036-WN	100 ng/ml
Prostaglandin E2	Tocris Bioscience	2296	1 µM
Y-27632	Sigma-Aldrich	Y0503	10 µM
Nicotinamide	Sigma-Aldrich	N0636	4 mM
A83-01	Tocris Bioscience	2939	0.5 µM
SB202190	Sigma-Aldrich	S7067	5 µM
HGF*	PeproTech	100-39	20 ng/ml

Table S2. Clinical baseline of patients included in this study from public TCGA-KIRC and ICGC-RCC cohorts.

Variables	TCGA (N = 537)	ICGC (N = 91)
**Age (Mean ± SD)**	60.59 ± 12.14	60.47 ± 9.97
**Follow-up (y)**	3.12 ± 2.23	4.14 ± 1.73
**Status**		
Alive	367 (68.34)	61 (67.03)
Dead	170 (31.66)	30 (32.97)
**Gender**		
Male	346 (64.43)	52 (57.14)
Female	191 (35.57)	39 (42.86)
**AJCC-T**		
T1	275 (51.21)	54 (59.34)
T2	69 (12.85)	13 (14.28)
T3	182 (33.89)	22 (24.18)
T4	11 (2.05)	2 (2.20)
**AJCC-N**		
N0	240 (44.69)	79 (86.81)
N1	17 (3.17)	2 (2.20)
Unknow	280 (52.14)	10 (10.99)
**AJCC-M**		
M0	426 (79.33)	81 (89.01)
M1	79 (14.71)	9 (9.89)
Unknow	32 (5.96)	1 (1.10)
**Pathological stage**		
I	269 (50.09)	-
II	57 (10.61)	-
III	125 (23.28)	-

IV	83 (15.46)	-
Unknow	3 (0.56)	-
**Grade**		
G1	14 (2.61)	-
G2	230 (42.83)	-
G3	207 (38.54)	-
G4	78(14.53)	-
Unknow	8(1.49)	-

Data are shown as n (%).

Abbreviations: TCGA, The Cancer Genome Atlas; ICGC, International Cancer Genome Consortium; AJCC, American Joint Committee on Cancer.
